# A mixed methods systematic review of cancer treatment decision-making in vulnerable populations

**DOI:** 10.1016/j.pec.2026.109491

**Published:** 2026-01-16

**Authors:** Charmaine L. Blanchard, Patricia McInerney, Maureen Joffe, Moosa Patel, Holly G. Prigerson, Shane Norris

**Affiliations:** a Strengthening Oncology Services Research Unit, Faculty of Health Sciences, University of the Witwatersrand, Non-Communicable Diseases Research Division, Wits Health Consortium, Johannesburg, South Africa; b Faculty of Health Sciences, University of the Witwatersrand, Johannesburg, South Africa; c Division of Haematological Oncology, Department of Medicine, Faculty of Health Sciences, University of the Witwatersrand, Johannesburg, South Africa; d Department of Radiology, Department of Medicine, Weill Cornell Medicine, Cornell Center for Research on End-of-Life Care, New York, New York, USA; e MRC/Wits Developmental Pathways for Health Research Unit, University of the Witwatersrand, Department of Paediatrics, Faculty of Health Sciences, Johannesburg, South Africa

**Keywords:** Mixed method review, Oncology, Patient treatment decision aids, Vulnerable persons, Low to middle income countries

## Abstract

**Introduction:**

Vulnerable cancer patients are at risk of making uninformed treatment decisions. Most patient decision aids (PtDAs) have been developed for high-income country (HIC) populations, with limited testing or adaptation for use in vulnerable populations, either contextually or geographically. This review aimed to understand how PtDAs address the treatment decision-making needs of vulnerable cancer patients.

**Methods:**

We conducted a mixed-methods systematic review of RCTs and other effectiveness studies of PtDAs as well as qualitative studies on the treatment decision-making experiences of vulnerable adults (≥18 years) with cancer. Eight databases were searched. Following PRISMA guidelines, we screened for eligible studies and appraised their methodological quality. Qualitative and quantitative findings were synthesized separately and then integrated to identify gaps and alignments between PtDA design and patient-reported decision support needs.

**Results:**

Twenty-six studies met the inclusion criteria (14 quantitative, 11 qualitative, and one mixed method). Qualitative synthesis showed that vulnerable patients experienced high emotional and psychosocial burdens, preferred in-person support, and often encountered mismatches between their preferred and actual decision-making roles. The narrative synthesis of quantitative studies showed that most PtDAs improved knowledge (5/7 studies) and reduced decisional conflict (6/7), while their effects on shared decision-making were mixed (3/5).

**Discussion:**

PtDAs that provided clear, concise information and were delivered face-to-face better addressed the needs of vulnerable patients than self-administered tools. Although patients reported significant emotional and psychosocial burdens, no PtDAs included structured counselling.

**Practice implications:**

Patient decision aids should be tailored to meet the informational, emotional, and role-support needs of vulnerable cancer patients, particularly in resource-constrained settings.

## 1. Background

Cancer treatment decisions are complex when there is clinical equipoise, with treatment options carrying varying risks of serious adverse events or functional loss. In such cases, well-informed decisions require not only adequate knowledge but also thoughtful deliberation, ensuring that choices align with the patient’s values and preferences [1]. The treatment decision-making process is influenced by the patient’s and physician’s backgrounds, personal characteristics, and communication skills. Vulnerable persons (for example, those with limited education, low health literacy, socioeconomic disadvantage, and cultural and linguistic differences [2]) face potential barriers to making informed decisions. Several frameworks define and stratify vulnerabilities within contextual, structural and individual domains from a broader geopolitical and economic perspective to life histories, social and individual vulnerabilities which exacerbate health inequities[2–4]. While patients in resource-constrained low- and middle-income countries (LMICs) may face distinct contextual vulnerabilities, underserved populations globally often encounter similar structural and individual-level barriers [5–10].

For all cancer patients, thoughtfully designed patient decision aids (PtDAs) that address their specific decision needs may improve knowledge and participation in cancer treatment decision-making without unduly increasing anxiety [11–15]. Most PtDAs have been developed and tested in high-income countries (HICs), with limited evidence of their use or adaptation in low- and middle-income countries (LMICs) [16,17]. While evidence exists of their effectiveness in supporting cancer screening decisions among vulnerable populations in HICs, evidence of the effectiveness of PtDAs in supporting treatment decision-making is limited [8,18]. This gap highlights the critical need for decision support interventions tailored to vulnerable persons, particularly in LMICs with constrained health and social systems, and among underserved populations globally. The Ottawa Decision Support Framework (ODSF) identifies key modifiable decisional needs that contribute to poor decision making such as knowledge limitations, decisional conflict, and lack of support, and recommends strategies such as clinical counselling, including establishing rapport and facilitating interactive communication, using PtDAs, and providing additional psychosocial support [1]. This framework has informed the development of numerous decision support interventions and provides a useful lens through which to evaluate the decision-making needs of vulnerable persons. However, few studies have clearly described the features of PtDAs that facilitate informed cancer treatment decision-making in vulnerable groups[8,9].

Given the limited evidence on the development and testing of cancer treatment decision support interventions in LMICs, we conducted a systematic review to assess the effectiveness of PtDAs in addressing the unique decision support needs of vulnerable patients in LMICs to inform the development of an intervention to support patients in making informed cancer treatment decisions. Our initial search was designed to identify studies conducted in LMICs. However, at this stage, no eligible LMIC studies were identified. Therefore, the review scope was extended to include studies from HICs that focused on vulnerable populations who may share similar decision-making barriers, with the intention of informing intervention development in LMICs. Thus, this review aimed to understand how PtDAs address the treatment decision-making needs of vulnerable cancer patients, regardless of whether they reside in an HIC or LMIC. The review questions were as follows: 1) What are the treatment decision-making experiences of vulnerable patients with cancer? 2) How effectively do PtDAs support the participation of vulnerable patients in informed cancer treatment decision-making?

## 2. Method

**Fig. 1.**
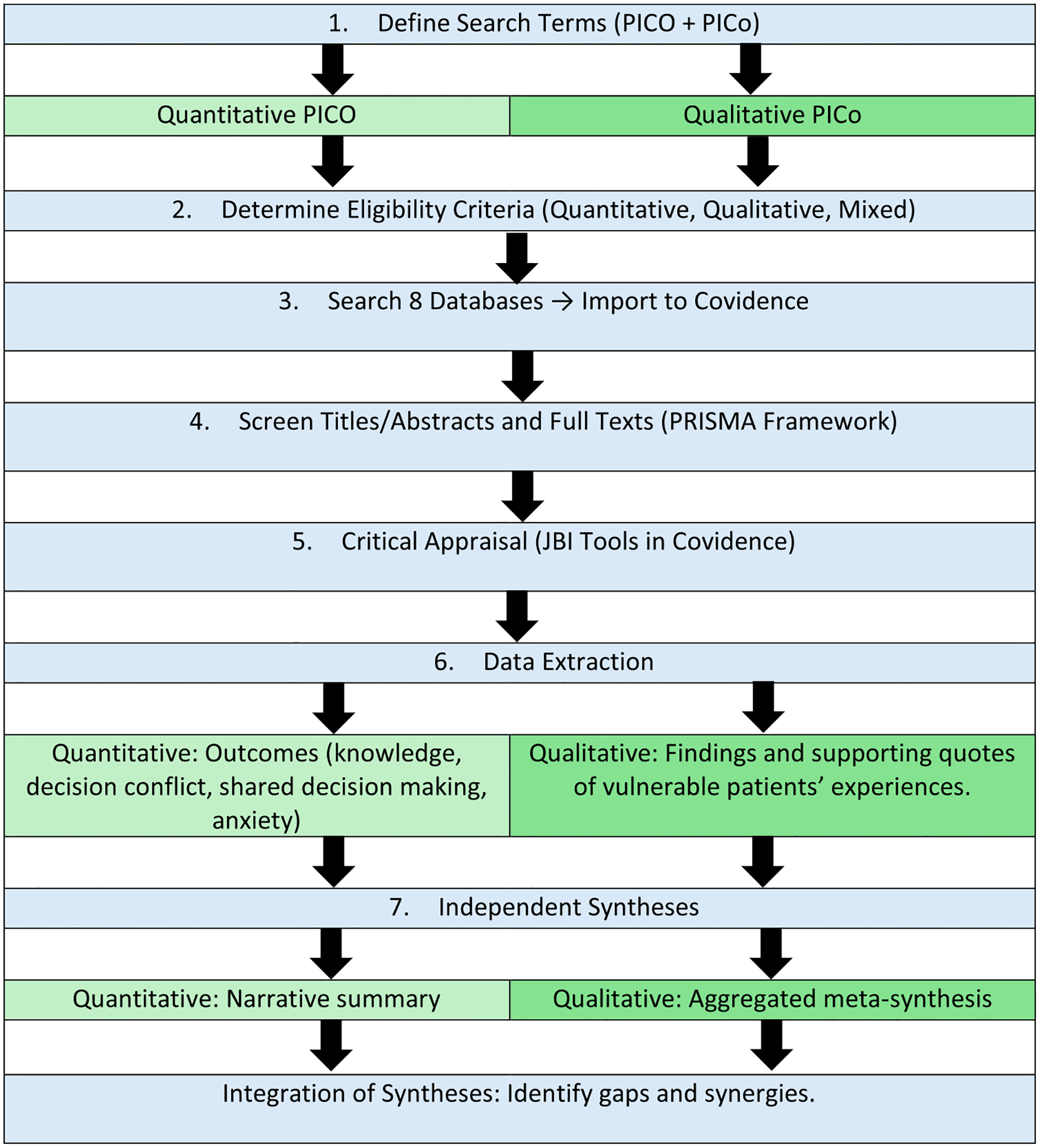
Overview of the convergent segregated mixed methods review process.

Following the Joanna Briggs Institute (JBI) methodology for mixed-methods systematic reviews, we used a convergent segregated approach for data extraction, synthesis, and integration of findings (Fig. 1) [19]. Given the complexity of cancer treatment decision-making, a mixed-methods review enabled a comprehensive understanding of how patient decision aids (PtDAs) address the decision support needs of vulnerable oncology patients and where gaps remain in both HIC and LMIC contexts [20].

### 2.1. Eligibility criteria

Using PICo (Population, Phenomenon of interest, Context) for qualitative data and PICO (Population, Intervention, Comparator, Outcome) for quantitative data, we included quantitative, mixed-methods, and qualitative studies of vulnerable patients (lower health literacy, limited education, socioeconomic disadvantage, ethnicity, race or language minority groups, or persons aged 65 years or older) aged ≥ 18 years diagnosed with adult-onset cancer, making cancer treatment decisions. Qualitative studies of decision-making experiences, with or without PtDAs, were included to explore decision-making needs even when no formal PtDA was present. In contrast, only quantitative studies evaluating the effectiveness of PtDAs among vulnerable patients—or those providing subgroup analyses for vulnerable populations—were included. Randomized controlled trials and non-randomized studies of PtDA effectiveness using control groups or usual care as comparators were included. Guided by the ODSF, quantitative studies were required to report at least one of the four predefined outcomes (knowledge, decisional conflict, shared decision-making, or anxiety/cancer-related distress). Studies reporting only other outcomes (e.g., regret, satisfaction) were excluded [1]. Mixed method studies were included if quantitative or qualitative data could be extracted separately. Owing to resource limitations, only papers published in English were considered. Because trials of PtDAs were first registered in the Cochrane database in 2000, concomitant with emerging literature findings in this field [21], we limited our searches from January 1, 2000, to April 30, 2024.

### 2.2. Search strategy and data sources

We searched the following databases: CINAHL, Global Health, MEDLINE, ProQuest, PsycINFO, PubMed, SCOPUS, and Cochrane Database. The JBI recommends searching the widest reasonable range of databases to ensure the review’s comprehensiveness. The search terms reflected four key concepts: (1) cancer, (2) treatment decision-making, (3) decision support tools, and (4) patient experiences and preferences (for qualitative papers). The terms were adapted for each database, and the full search strategies are presented in Supplementary Table A.1. The initial search included studies from January 1, 2000, to September 30, 2021. The search was intentionally broad and did not include terms specific to LMICs to maximize sensitivity and avoid excluding potentially relevant studies. However, this search yielded no studies conducted in LMICs. We therefore expanded our search to include terms related to vulnerable or disadvantaged persons, recognising overlap in contextual decision-making challenges (full search terms in Table A.1.) To ensure that no LMIC-based studies were inadvertently missed in the original search, we undertook an updated tiered search strategy.

1. We replicated the original broad search from October 01, 2021, to April 30, 2024.
2. We re-ran the original search strategy with LMIC-related terms for both the original and updated search periods solely to identify any LMIC studies that may have been missed.
3. We conducted a third search combining terms related to vulnerable populations with the original search strategy (without LMIC terms), applied to the updated period only.

We conducted a citation search of relevant papers to identify additional papers and identified additional papers from websites for inclusion in the review.

### 2.3. Study selection and appraisal

We implemented the Preferred Reporting Items for Systematic Reviews and Meta-analyses (PRISMA) guidelines [22] for study selection and inclusion. The search results were imported into COVIDENCE© to manage the deduplication and screening of titles, abstracts, and full texts [23]. Five reviewers screened titles and abstracts using inclusion/exclusion criteria, with study inclusion decided by the consensus of two reviewers; conflicts were resolved in weekly meetings. The full texts were screened in a similar manner. Two independent reviewers appraised the methodological quality using JBI standardized tools [24]. (8–13 questions; Supplementary Table A.2). without applying a cutoff score. Disagreements were resolved through discussion without the need for a third reviewer.

### 2.4. Data extraction

We extracted details of the PtDAs and study outcomes (knowledge, decision conflict, shared decision-making, and anxiety) from quantitative studies, and themes and subthemes with supporting quotes from qualitative and from mixed method studies. We accessed additional details of the interventions from the cited papers describing intervention development.

Two reviewers independently extracted data for the original review and resolved disagreements through discussion. For the update, one reviewer extracted the data, and a second cross-checked the critical appraisal and extraction through discussion, based on prior calibration and familiarity with the extraction domains established in the original review.

### 2.5. Data synthesis and integration

Utilizing the JBI convergent segregated approach [24], we synthesized the quantitative and qualitative evidence separately, using an integrative matrix to compare vulnerable patient experiences with PtDA outcomes. Due to heterogeneity, meta-analysis was not feasible, and quantitative results were summarized narratively. Qualitative findings were pooled and categorized following the JBI meta-aggregation. The aggregated results were juxtaposed to identify synergies and gaps between patient experiences and PtDA outcomes.

## 3. Results

**Fig. 2.**
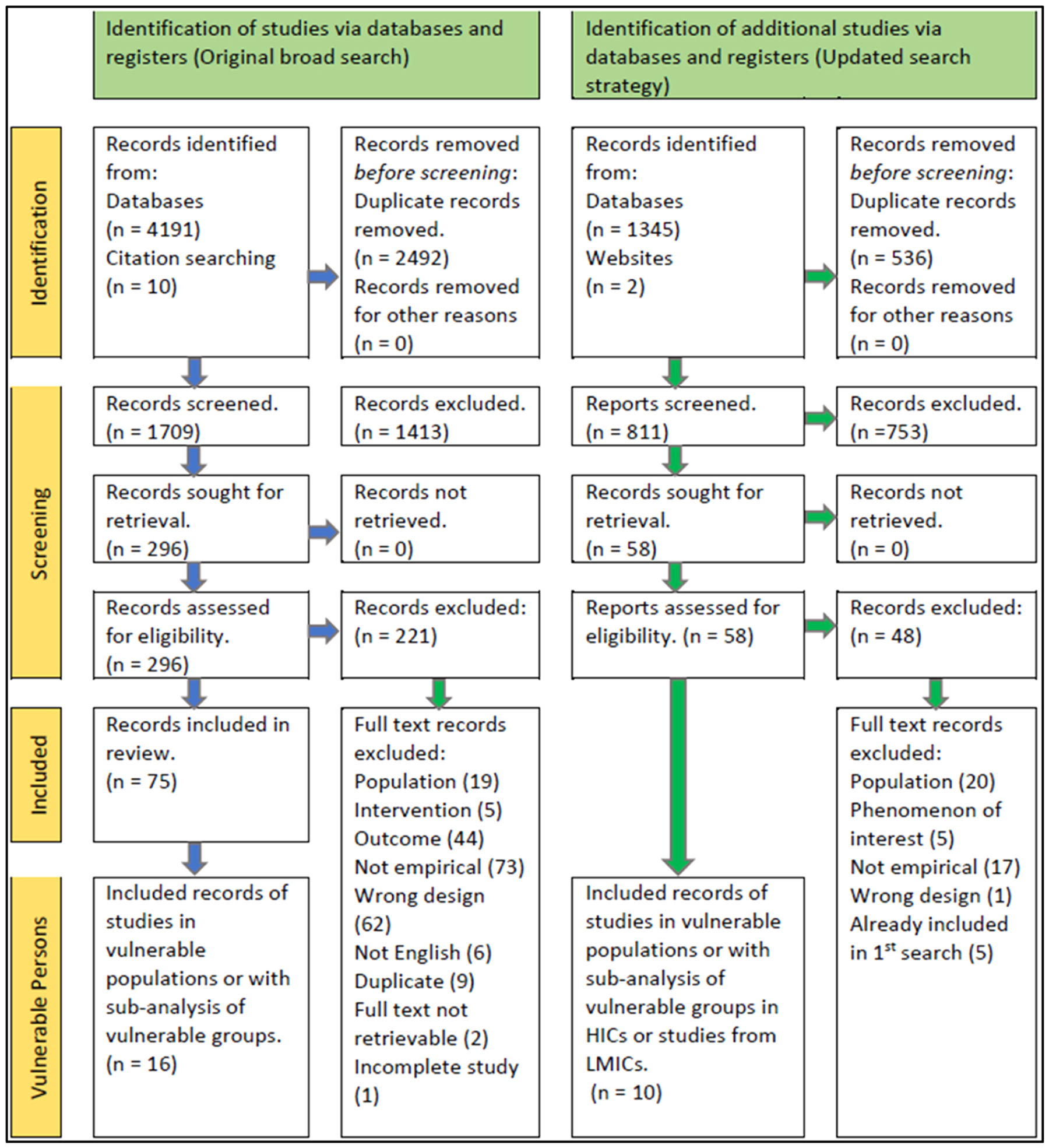
PRISMA flow diagram of study selection. (PRISMA = Preferred Reporting Items for Systematic Reviews and Meta-Analyses) [22] (HICs = high-income countries. LMICs = Low- and Middle-Income Countries.).

The first broad search (01 January 2000 – April 30, 2024) yielded 4191 papers, of which 2492 duplicates were removed. Citation screening yielded 10 papers. Of the 1709 papers screened, 16 were eligible based on our inclusion criteria for vulnerability (Fig. 2) [22].

Our updated database search in May 2024 yielded 1345 papers for screening, resulting in 10 eligible papers. From both database searches, 26 papers were included (14 quantitative, 11 qualitative, and 1 mixed method, from which we extracted qualitative data).

### 3.1. Methodological quality of studies

The 11 qualitative and one mixed-method paper scored ≥ 6/10, with most (11/12) demonstrating good alignment between objectives, methodology, and interpretation. However, 58.3 % of the papers lacked a clear theoretical framework, only 33.3 % reported researcher positionality, and 91.6 % did not address reflexivity, raising the potential for bias. Among the 12 randomized controlled trials, 91.7 % reported true randomisation, but 83.3 % did not specify blinding of participants, clinicians, or outcome assessors, limiting internal validity.

### 3.2. Overview of the studies

#### 3.2.1. Qualitative and mixed method studies

Of the 12 papers 2 qualitative papers reported results from the same study [25,26] Alam et al. (2016) and Durand et al. (2016) reported different aspects of a development study of the Option Grid (OG) and Picture Option Grid (POG) PtDAs. Both papers drew on the same participant sample but addressed different objectives. Of the 10 qualitative studies and one mixed method study [27], nine (81.8 %) were conducted in HICs: five in the USA [25,26,28–31], two in the UK [32,33] and one each in Canada [34] and Australia [35].(Table 1). Two papers (16.7 %) reported on studies from an upper middle-income country (China) [27,36].

Data collection methods included individual participant interviews [25–28,31,32,36,37], focus group discussions [30,34], or both [35]. Six (54.5 %) studies focused on women with breast cancer [25,26,28, 30–32,34], three (27.3 %) on men with prostate cancer [29,33,36], and two (18.2 %) on patients diagnosed with any cancer [27,35]. The detailed study characteristics are shown in Table 2.

#### 3.2.2. Quantitative studies

Fourteen quantitative papers reported results from 13 studies. Two papers (Durand 2021; Yen 2020) reported findings from the same RCT evaluating the (OG) and (POG) PtDAs, and therefore contribute a single dataset to the synthesis [38–50] (Table 1). Two papers (Berry 2013 and 2018) evaluated the same PtDA (Personal Patient Profile–Prostate) in independent patient cohorts. Nine (64.3 %) quantitative studies were conducted in HICs [38–41,44–49], with the majority (n = 7, 53.8 %) in the USA [38–40,44–47,49] (Table 1). Four (28.6 %) studies were conducted in LMICs [42,43,50,51]. The 13 studies comprised 11 RCTs (84.6 %) [38–48,51], one analytical cross-sectional study [49], and one quasi-experimental study [50]. Two RCTs evaluated two PtDAs, resulting in 14 PtDAs being included in this review. [38,44,46] Six (42.8 %) studies focused on localized prostate cancer [39,40,42,44,45,49], five on localized breast cancer [38,43,46–48,51], and one each on hepatobiliary cancer [50] and metastatic colorectal cancer [41]. The detailed characteristics of the quantitative studies are listed in Table 3.

### 3.3. Qualitative synthesis of studies of patients’ experiences

The 11 qualitative and mixed-methods paper yielded 84 findings, comprising the original themes and subthemes of the articles included in the review. Alam (2016) and Durand (2016) report different thematic analyses from the same development study [25,26]. Although some concepts overlapped, each paper presented unique themes and supporting participant quotations; therefore, all findings were extracted and assessed during synthesis. The 84 findings were aggregated into 13 categories based on theme similarities. The 13 categories were grouped into four major synthesised findings, as summarized in Table 4. A detailed description of the synthesized findings with themes from the original findings and excerpts from the reviewed articles is provided in Supplementary Table A.3.

#### 3.3.1. Synthesised finding 1: Patients preferred individualised, easy-to-understand information provided personally by healthcare providers

This finding comprises three categories: 1) information needs, 2) information gathering, and 3) information content and format.

1. Patients need clear information about their cancer and prognosis, available treatments, and behaviour during treatment. “*she (the patient) wanted to know “what it is and what your options are in a very straightforward way. I don’t think you should be kept wondering”.”* [34]
2. Patients usually relied solely on clinicians for information, even when they felt confused during the consultation. Few would search the Internet, *“The doctor said clearly, and the effect is very obvious. We checked it online and learned something”* [27] while some would speak to other doctors or family *“No the doctor first tell me you need this… and then I asked the other doctors and they say this is the best so of course I come back and say I want to do it because they all say it’s the best.”* [35]
3. Concise, individualized information with simple, straightforward text, pictures, or videos is preferred. “*And sometimes when people explain things it sounds different but to see the pictures and to have an idea of what actually was happening, I think would help a lot of people”* [26]. Older or less-educated patients felt less comfortable with Internet-based information. “*I’ve got it, [internet] but I don’t bother about it very much … No, it would be the last thing I’d do [go to the internet]”* [32].

#### 3.3.2. Synthesised finding 2. Decision making is a process that requires time and effort to consider options under stressful circumstances, and decisions do not always reflect the best clinical choice

This synthesized finding comprises the following categories: (1) process and timing, (2) deliberation on options and choices, and (3) reasons for choices.

1. Patients felt the process was rushed without enough information at the time to make decisions, “*things have to be decided too fast’….’lack of knowledge’ … ‘just didn’t feel sure”* [28], and receiving a DA prior to the consultation was preferred, *“I’d like to have something like this in the mail before I go and see my surgeon.”* [26]
2. Making decisions was effortful for patients requiring personal direction and support, *“the navigator helped her to ‘get in the habit of writing questions and reviewing them before seeing her doctor.’”* [30] They felt they lacked the knowledge to participate in deliberating options in the consultation: “*I do not think I should be involved in this process. I don’t know about pharmacology. I believe in doctors”* [27], some relying on vicarious experiences to make decisions, *‘I know what [chemotherapy] did to my husband. It took all of his hair out.’* [31] Patients appreciated having PtDAs to inform their decisions, “*In all the pages, it shows that you either take part of your breast or all of your breast, then it shows you how long it takes, then it shows you the radiation, so I know what’s coming…that was my fear, what is coming.”* [26]
3. While survival is a key motivator, treatment choices may be determined by social and emotional factors rather than the most clinically appropriate choice. *‘I was just going to have to leave it [the cancer] go. ‘ (had no insurance)* [28].

#### 3.3.3. Synthesised finding 3. Clinician–patient communication impacted decision-making consultations, and patients did not always adopt their preferred decision roles; however, decision support aids enabled more active engagement

Four categories were related to the consultation process: 1) clinician-patient interaction, 2) preferred or perceived passive decision-making role, 3) preferred or perceived active decision-making role, and 4) decision support in the consultation.

1. With a few exceptions, patients felt unheard during unidirectional oncologist-patient communication. “*I think that was the worst part of my whole breast cancer surgery…Nobody was listening to me”* [28]. Patients felt more supported when they had time to talk to their health caregivers, *“I have excellent communication with [providers]. They explain things. They take time with me. They always call me back”* [31]. Language barriers limit patient participation in consultations. *“I can read English, but I don’t understand the meaning of the words. I have to take out the dictionary every time I come from the doctor’s. I just sit there and say yes, yes but I leave his consultation and I didn’t understand most things. I find it hard” (Arabic patient)* [35].
2. Many patients preferred a passive decision-making role, trusting the doctor to know what was best for them *“… I just feel that it’s not up to me to decide, I am not a professional doctor, so I have to rely on the doctor, whatever he tells me to do.”* [35]
3. Few patients adopted an active decision-making role and some resented the passive role they felt compelled to accept, *“Since I have been in the hospital, I have been instructed to undergo various checks or tests. It’s as if I were a puppet. I want to be able to decide whether or not to do these checks.”* [36]. Feeling disempowered to actively participate was distressing: *“For my case, the specialist never discussed with me, never took my opinion in consideration… The doctor won’t listen to you. It’s not that we didn’t want, and it’s not that we didn’t initiate to know more, I really wanted to know, I felt helpless, felt myself helpless. He “HOLD” the “POWER”.”. ’ (emphasis from the original paper)* [35].
4. Simple PtDAs promoted more active participation in the consultations. “*It leaves room for you to ask questions. I mean it puts things into your*—*into perspective for you to understand more and to be more comfortable asking your doctor, I think.”* [26], and the presence of family members facilitated interactive discussions. “*I had two of my daughters with me, and they asked a lot of questions. But since I’ve been going by myself, I [have] really felt neglected”* [31].

#### 3.3.4. Synthesised finding 4. For newly diagnosed patients, in addition to social, cultural and language factors, strong emotions influenced decision making, indicating the need for an enhanced psychosocial approach to decision support

This finding comprised three categories: 1) the emotional impact of cancer diagnosis, 2) family, social, and cultural influences on decision making, and 3) strategies for socio-emotional support.

1. Upon receiving a cancer diagnosis, patients felt emotionally over-whelmed, reducing their ability to rationally consider treatment options. “*The problem was I wasn’t deciphering [the information] because I was so afraid”* [28].
2. Family dynamics influence treatment deliberation and choices. Family roles in decision-making varied from not playing any role: “I *do have family, but they don’t say much. They do not give me much information. My family knows about the problem, but they only say for me to take care. Nothing else.”* [29], to complete decision control, “*I don’t know how children and doctors negotiated. They let me treat here first, and then went back to surgery. I asked if I couldn’t do the surgery. The kid said no, I had to do the surgery. I do not have a choice”* [27]. Cultural and religious beliefs affect treatment discussions, decisions, and adherence. *“I take Chinese herb; I asked my doctor. The doctor didn’t agree with me at the beginning. For example, during the chemotherapy, the doctor told me I shouldn’t take anything, but I felt it would be better. I took Ling-Zhi and shark bone powder now.”* [35]
3. Patients wished for emotional support, “*It would have been helpful to meet with a social worker or psychologist to help them come to terms with “stresses they are going through”* [34]. Talking to cancer survivors, patient navigators, or decision coaches not only helped with understanding information but also provided emotional support. “*I appreciated speaking with her (the cancer survivor coach). She made me feel quite at ease in the face of a difficult situation. She gave me hope”* [31]

### 3.4. Quantitative synthesis of effectiveness of decision support interventions

Twelve PtDAs were self-administered at home or in the clinic [39–45,47–51], with two reporting staff assistance [47,49]. Of the self-administered PtDAs, nine were accessible online [39,44,45,47–51], three were paper-based [42,43,48]. Six PtDAs included videos [39,41, 44,45,47,50], of which only one was not available online [41]. Six PtDAs were administered in consultation with an oncologist [38,43,44, 48,50,52], of which one provided a summary for the patient [44]. No PtDAs included counselling or coaching by a dedicated counsellor; however, one study reported that a case manager assisting patients with self-administered PtDAs provided emotional support as required [47]. The narrative synthesis of effectiveness outcomes comprised four groups: knowledge, decision conflict, shared decision making, anxiety, and cancer-related distress. (Detailed findings are presented in Table 4)

#### 3.4.1. Knowledge Outcomes

Of the seven studies assessing participants’ knowledge of cancer and treatment [41,42,44,46–48,50], five demonstrated a significant increase in knowledge with PtDA use [42,46–48,50] (Table 2). AlSagheir *et al*. found that low income and a lower level of education had no impact on patients’ understanding of cancer and treatments [41]. Durand et al. reported that the Picture Option Grid (POG) PtDA reduced disparities in knowledge between patients with lower and higher SES more than usual care (UC) [46]. The POG also improved more outcomes than the Option Grid (OG) PtDA, possibly because of the pictorial content, as reported by the investigators.

#### 3.4.2. Decision conflict outcomes

Berry et al. (2018) found that their PtDA significantly decreased decision conflict at one month, but that greater decision conflict was associated with unemployment and lower income [40]. In an earlier study, Berry *et al*. (2013) reported no significant decrease in the total DCS score over time (baseline to six months); however, the sub-scores for uncertainty and values clarity improved significantly [39]. Older age, being non-White, having a lower income, having more trait anxiety, and having inadequate baseline information and support were associated with being less informed (subscale). A higher total DCS score was associated with a lack of support and uncertainty at baseline, clinic site, and shorter time from enrolment. While Diefenbach *et al*. found no impact of their PtDA on total DCS scores, perceived decisional support was increased, and African Americans and persons with lower education reported greater levels of support after using the PtDA [45].

#### 3.4.3. Shared decision-making outcomes

Four studies reported SDM outcomes of five PtDAs [38,43,46,48,49], of which three showed a positive effect [38,46,49]. One RCT demonstrated improved SDM outcomes with 2 PtDAs [38,46], while in a cross-sectional study Li et al. reported high levels of SDM with no difference between study sites, one of which served a largely vulnerable population [49]. Durand et al. reported that the in-consultation OG and POG PtDAs resulted in greater SDM than UC but that the POG resulted in greater levels of self-reported and observed SDM [46]. Yen et al. reported similar results from the same RCT, but that the observed SDM scores were significantly lower for people with low SES but not for those with low health literacy [38].

#### 3.4.4. Anxiety and cancer related distress outcomes

Three studies measuring anxiety or cancer-related distress after the decision-making consultation reported no significant differences between the intervention and control groups [41,45,48].

### 3.5. Integration of the syntheses of qualitative studies of patient experiences and quantitative studies of decision aid effectiveness outcomes

The four synthesized findings of patient experiences were matched to the outcomes of effectiveness studies (Table 5).

#### 3.5.1. Knowledge outcomes and patient-reported information needs, content, format, and sources

Vulnerable patients seek information from their doctors, family, and friends, with few accessing information from the Internet. They preferred pictures, videos, and printed materials with minimal simplified text. Of the five PtDAs that improved patients’ knowledge [42,46–48,50], four presented information in pictures or video format [46–48,50], three were used in consultations [46,48,50], and one was used with the assistance and emotional support of a case manager [42]. Two of the three PtDAs that did not improve knowledge were self-administered [41,44]. PtDAs that addressed patients’ needs for simple, understandable information with support from clinic staff were more effective in improving their knowledge of cancer and/or treatments.

#### 3.5.2. Decision conflict (DC) scores and patient-reported decision-making needs

Patients who felt pressured to make decisions and deliberated on options without sufficient information appreciated having decision support. Their treatment choices are often influenced by contextual factors. All seven PtDAs reduced total decision conflict or its subscales. They included values-clarifying exercises [39,45,47,49–51], four provided decision coaching [39,45,47,50], and three provided a summary page for patients and/or physicians [39,47,49]. All were self-administered, with one included in the consultation [50]. Despite their differing formats, PtDAs address most decisional conflict needs.

#### 3.5.3. Shared decision making (SDM) and decision control preferences

Vulnerable persons reported difficulties engaging with doctors during the consultation, and a passive role was commonly adopted, even if active participation was preferred. Of the four PtDAs assessed in three RCTs, two significantly improved SDM compared to controls [38,46,48], while a PtDA assessed in an analytical cross-sectional study resulted in high levels of SDM (mean scores of 36 and 42 out of 45) [49]. Of the in-consultation PtDAs, two paper-based tools and a staff-assisted self-administered PtDA with reports for the patient and clinician improved SDM [38,46,49]. A question prompt list (QPL) provided to patients to prepare questions prior to the consultation did not improve SDM [43]. Patient decision aids that improve oncologists’ engagement in SDM may improve patient participation in decision making more than preparing patients alone.

#### 3.5.4. Anxiety and other emotional and sociocultural factors impact decision-making

Patients consistently mentioned the emotional impact of receiving a cancer diagnosis, which affected their ability to make decisions, with some expressing a preference for additional in-person counselling support that extended beyond the clinical consultation. A self-administered PtDA that included a distress-lowering exercise had no effect on anxiety post-intervention [45]. However, it is unclear whether PtDAs using vignettes matched to patient subgroups for decision coaching address cultural and social influences on choices. An Indian trial of a culturally adapted PtDA included a second intervention arm with patient-caregiver dyads, resulting in reduced decision conflict [51]. No trials of PtDAs specifically reported professional counselling (outside of the clinical consultation) to address emotional, social, cultural, and religious influences on decision making.

Table 6 presents a matrix of patients’ decision support needs and PtDA characteristics to identify gaps and synergies between decision needs and elements of the PtDAs.

Abbreviations: DA, decision aid; SDM, shared decision making; P3P, personal patient profile prostate; OG, option grid; POG, picture option grid; CPtDA, computerised multimedia interactive patient DA; DESI, decision support intervention; PPT, patient preference tool; PDA, patient DA; QPL, question prompt list; NSD, no significant difference between control and intervention; ↑/↓, outcome significantly greater/less than control (positive effect); NSD, no significant difference in outcome between intervention and control; **√,** included in PtDA. * Durand and Yen report findings from the same RCT

## 4. Discussion and conclusion

### 4.1. Discussion

This mixed method review integrated qualitative and quantitative evidence to examine how PtDAs support treatment decision-making among vulnerable cancer patients in HICs and LMICs. Overall, PtDAs improved knowledge, reduced decisional conflict, and enhanced SDM without increasing anxiety. These outcomes are broadly consistent with the most recent Cochrane review, which reports similar benefits across diverse populations. [53] However, our synthesis additionally shows that PtDA effectiveness is strongly influenced by whether tools address vulnerable patients’ informational, relational, and contextual needs. Many interventions only partially meet these needs, limiting their real-world impact. Reviews focusing on health-related decision-making experiences of disadvantaged populations describe persistent challenges in accessing understandable information, navigating emotionally demanding decisions, and participating in SDM, patterns mirrored in our findings [17,54–57]. Similar to Yen et al.’s meta-analysis of socially disadvantaged groups, we found that simplified formats, pictorial information, and staff-supported delivery improved outcomes but did not fully eliminate inequities in decision participation [58]. Our findings reinforce and extend this literature by identifying specific PtDA components: value-clarification exercises, tailored visuals, clinician-facing summaries, and decision coaching, that better meet the decision-making needs of vulnerable patients in cancer care.

Patients reported varied preferences for the type, amount, and format of information, and many relied on clinicians rather than on online sources. PtDAs using pictures, videos, or narrative elements improved knowledge more consistently than text-based tools, especially when integrated into consultations or when supported by staff. This reflects both the Cochrane review’s conclusion that PtDAs improve knowledge and other oncology reviews highlighting the utility of visual formats [18,53,59]. However, our findings show a stronger reliance on interpersonal and clinician support among vulnerable groups, indicating that static, self-administered PtDAs alone may be insufficient when literacy or contextual barriers are significant.

Decision-making occurred under emotional strain, influenced by fear, urgency, and limited decisional support. While most PtDAs reduced decisional conflict, emotional distress, and limited support remained influential, similar to findings of a review of PtDAs for older adults, which identified emotional burden as a barrier to effective decision-making [55]. Videos with relatable stories improved comprehension but did not fully address the emotional effort involved in the process. Few interventions incorporated counselling or structured decisional support despite ODSF recommendations, highlighting a notable evidence gap in this area.

Vulnerable patients frequently described challenges in engaging in SDM, including limited confidence, communication barriers, and norms favouring clinician- or family-led decisions, findings consistent with other reviews [54,55,60–63]. In our synthesis, PtDAs integrated into consultations or clinician-facing summaries improved SDM more effectively than patient-only preparation tools. This aligns with the Cochrane review’s finding that PtDAs can enhance participation [53] however clinician engagement appears to be a key factor influencing PtDA effectiveness in vulnerable populations. A question prompt list alone did not improve SDM, suggesting that preparing patients without changing clinician behaviour is insufficient.

Cultural norms, family roles, religious beliefs and socioeconomic pressures influenced decision-making. At times contrary to cultural norms, some patients felt excluded by family-led decisions, while others found family involvement supportive, similar to a review emphasizing the importance of individual experiences [64]. Decisions did not always align with clinical recommendations, possibly being influenced by personal and socio-economic factors [47,48], as reported in a review highlighting novel contextual influences on decision making [17]. Although culturally tailored videos were helpful, no included PtDAs provided structured psychosocial, cultural, or family-based counselling. This gap is consistent with prior reviews, highlighting the need for PtDAs that incorporate relational and cultural dimensions rather than focusing solely on information provision [8,55].

Although most evidence came from HICs, similarities in decisional needs across settings supported the inclusion of these studies. While methodological limitations, such as inconsistent reporting of randomisation procedures and limited reflexivity, were present, overall confidence remains moderate to high because of the convergence of findings across studies. The findings are likely transferable to vulnerable groups facing preference-sensitive decisions. However, the evidence from LMICs remains limited to a narrow set of cultural contexts. Importantly, few PtDAs comprehensively addressed emotional, cultural, and contextual influences, mirroring the limitations noted in earlier oncology and vulnerable population reviews.

This review was limited to English-language studies, potentially missing relevant research from non-English-speaking countries, including LMICs. Quantitative studies varied in terms of intervention delivery and outcomes, complicating comparisons. However, integrating patient-reported needs with effectiveness data offers novel perspectives on the features of PtDAs which will potentially support informed cancer treatment decision making for vulnerable persons across all settings.

The findings reinforce that vulnerability, driven by low literacy, socioeconomic disadvantage, or structural barriers, impacts decision-making across contexts. Implementation, particularly in LMICs should consider available resources and embed PtDAs into clinical workflows, incorporating interpersonal support and using task-shifting approaches for counselling and decision coaching [65–67]. Consistent with ODSF guidance and recent reviews, PtDAs must integrate emotional, social, and cultural support to be fully responsive to vulnerable patients’ needs.

### 4.2. Conclusion

Vulnerable patients across diverse settings face persistent barriers to participating in informed cancer treatment decision-making processes. PtDAs can improve knowledge, reduce decisional conflict, and enhance SDM; however, their effectiveness depends on tailoring to literacy needs, addressing emotional and cultural contexts, and integrating support into clinical encounters. Approaches that combine clear information, interpersonal support, and facilitation of role preferences are likely to be the most effective in both HICs and LMICs.

### 4.3. Practice Implications

To support vulnerable patients in HICs and LMICs, PtDAs should:

1. Use clear, visual, and tailored information appropriate for diverse literacy needs.
2. Be integrated into clinical workflows to encourage clinician engagement in SDM.
3. Address the emotional, cultural, and social influences on decision-making.
4. Incorporate task-shifting approaches for counselling and decision coaching.
5. Adapt effective strategies from HICs, considering LMIC resource constraints.

## Supplementary Material

1

2

3

## Figures and Tables

**Table 1:** Overview of study characteristics.

Study characteristics		
	Qualitative and mixed method studies (n 11)	Quantitative studies (n 13)
	n (%)	N (%)
**Country**		
USA (HIC)	5 (45.5) *	7 (53.8) *
Saudia Arabia (HIC)	-	1 (7.7)
UK (HIC)	2 (18.2)	1 (7.7)
Australia (HIC)	1 (9)	-
Canada (HIC)	1 (9)	-
China (UMIC)	2 (18.2)	1 (7.7)
Malaysia (UMIC)	-	1 (7.7)
Iran (LMIC)	-	1 (7.7)
India (LMIC)		1 (7.7)
**Study design (Quantitative studies)**		
RCT	-	11 (84.6)
Quasi-experimental	-	1 (7.7)
Analytical cross-sectional	-	1 (7.7)
**Data collection methods (Qualitative (10) and mixed method (1) studies)**		
Semi-structured interviews	8 (72.7)	-
Focus group discussions (FGDs)	1 (9)	-
Semi-structured interviews + FDGs	2 (18.2)	-
Sample size		
< 100	11 (100)	3 (23.1)
101–400	0	8 (61.5)
> 400	0	2 (15.4)

Two qualitative papers reported on one study and two quantitative papers reported on one study

**Table 2:** Characteristics of the 11 qualitative and 1 mixed method papers.

Lead Author, Date	Title	Method	Phenomena of interest	Geographic, Setting	Cultural	Participants
Alam, S 2016 USA	Assessing the acceptability and feasibility of encounter decision aids for early-stage breast cancer targeted at underserved patients	Semi-structured interviews (Phase 3 of a multi-phase study).	Acceptability and feasibility of three encounter decision aids (one text-based encounter decision aid and two pictorial encounter decisions aids) targeted at a low SES and low literacy population, using plain language and images.	Dartmouth-Hitchcock Medical Centre (Lebanon, NH) and Chelsea HealthCare Centre (Chelsea, MA). Upper Valley in New Hampshire and Vermont, USA.	Not stated, except that low SES (on Medicaid or Medicare without supplemental insurance or without health insurance – as proxy for low SES) groups in the Upper Valley in New Hampshire and Vermont, USA.	10 women of low SES who had been diagnosed with early-stage breast cancer in the past five years. The mean age was 56.8 years (SE 4.40).
Bamidele, O 2021 UK	A constructivist grounded theory study on decision-making for treatment choice among Black African and Black Caribbean prostate cancer survivors	Semi-structured Interviews	Treatment decision-making among BA and BC men as influenced by a CaP illness diagnosis and a unique socio-cultural context	Residents of England	Black African or Black Caribbean ethnic origins residing in the UK.	8 BA and 17 BC men, average age 65 years (50–88 years). Education levels: at least secondary school (n = 13), graduate (n = 5) or post graduate (n = 5) levels. 23 men had been resident in the United Kingdom (UK) for over 20 years.
Burton, M 2015 UK	The information and decision support needs of older women (>75yrs.) facing treatment choices for breast cancer: a qualitative study	Semi-structured Interviews	Information needs and preferences for the older age group (>75yrs.) of women relating to the choice between surgery and PET	UK Clinics	Not stated except that these were older women (>75 yrs.) who had been diagnosed with invasive breast cancer in the preceding 60 months and offered a choice of PET or surgery.	33 women with age range of 76–91 years; 22 women received PET and 11 underwent surgery.
Durand, 2016 USA	‘Much clearer with pictures’: using community-based participatory research to design and test a Picture Option Grid	Semi-structured Interviews	Usability, acceptability and accessibility of a pictorial encounter decision aid targeted at women of low SES diagnosed with early-stage breast cancer	Underserved community settings (e.g., knitting groups, bingo halls, senior centres) and breast clinics in USA.	Underserved community settings (e.g., knitting groups, bingo halls, senior centres) and breast clinics in USA.	10 women of low SES with stage 1 - stage 3 breast cancer. Mean age 56.8 (range 31–75)
Huang S 2022 China	Status quo of advanced cancer patients participating in shared decision-making in China: a mixed study	Mixed method for individual interviews for qualitative enquiry.	Patient’s views on SDM and factors affecting their participation (including the intrinsic reasons and patient motivation) in SDM.	16 hospitals from 9 provinces of China	Chinese cultural approach to consultations with doctors.	17 tertiary hospital patients with stage 3 or 4 cancer, expected survival ≥ 3 months. Age range 35 – 76, 8 female, education range primary to high school, 8 rural medical insurance, 8 unemployed.
Mcvea, KLSP 2001 USA	Low-income women with early-stage breast cancer: Physician and patient decision-making styles	In-depth interviews	Previously unsuspected factors influencing cancer decision making in real world settings	USA State of Nebraska indigent patient assistance program.	Mainly white indigent women.	Women (25 in total) with stage 1 or 2 breast cancer. Median age 56 years, 19 rural-dwelling, 22 white, 3 non-white.
Michel, J 2021 USA	Improving Shared Decision Making in Latino Men with Prostate Cancer: A Thematic Analysis	Semi-structured in-depth telephone interviews	Prostate cancer patients’ information gathering, preference for information presentation, technology use and to identify qualities of ideal decision-making treatment.	USA Metropolitan medical centre urology clinic.	Underinsured low-income Latino population in Los Angeles. Individuals originally from South America, Central America, and Mexico.	20 men recently diagnosed with prostate cancer age 50 or older (range 52–74), in the process of deciding on a cancer treatment. Formal education 8.6–11.9 years.
Pan S 2022 China	Patient participation in treatment decision-making of prostate cancer: a qualitative study	Semi-structured interviews	Decision-making perceptions and experiences of prostate cancer patients	Urology department of an A-level tertiary teaching hospital in China.	Chinese participants	30 men Age, mean (SD) 67,2 (6.96) Education: Elementary school or less: 10 (33.3 %) Tertiary: 4 (13.3 %) Monthly income: RMB ≤ 8000: 20 (66.6 %)
Shaw, J 2015 Australia	Treatment decision making experiences of migrant cancer patients and their families in Australia.	Focus group discussions and individual semi-structured interviews	Factors that influence the cancer treatment decision-making experiences of first-generation migrants with cancer from Arabic, Chinese, or Greek backgrounds.	Australia Community-based cancer support groups and three oncology outpatient clinics.	First generation migrant from a Chinese (Cantonese or Mandarin), Greek or Arabic speaking country, and had one of these languages as their first language.	Patients 18 years and over who had been diagnosed with cancer or been a cancer patient caregiver in the preceding 3 years.
Sheppard, VB 2008 USA	Latina a Latina (SM): developing a breast cancer decision support intervention.	Focus groups sessions	Patients’ perceptions and experiences making treatment decisions and survivor advocates’ breast cancer stories	USA Patients from support groups and physicians, patient advocates from a Latino breast cancer resource organisation.	Persons from the Latin American population (including islands of the Caribbean) as well as Latin Americans who speak Portuguese, Quechua, and Guarani.	Women with breast cancer and breast cancer survivors most from Central America and age range 34–59.
Sheppard, VB 2010 USA	Development of decision-support intervention for Black women with breast cancer	Semi-structured, In-depth interviews	Factors that influence Black women’s′ adjuvant therapy decisions	USA Washington DC metro area.	Black women patients (14) who were residents of the District of Columbia. Survivor advocates (10), and cancer providers (total 10–5 Black, 3 White, 2 Asian).	Breast cancer patients in active treatment, survivor advocates who mentored other women, and cancer providers.
Wong, J.J 2011 Canada	What Do Older Patients with Early Breast Cancer Want to Know While Undergoing Adjuvant Radiotherapy?	Focus groups discussions	The experiences of women with breast cancer, diagnosis and treatment	Canada Radiation therapy clinic.	Not stated, other than English speaking.	Breast cancer patients, over the age of 70years.

**Table 3:** Characteristics and outcomes of the 14 intervention papers.

Lead Author, year, Study design Country of study	Title of paper	Description of the interventions	Setting and study population	Outcome instruments	Outcomes
Aeshah I. AlSagheir 2020 RCT Saudi Arabia	Comparing the use of Arabic decision aid to usual care. A multicentre randomized controlled trial for Arabic speaking metastatic colorectal cancer patients in Saudi Arabia	**Description** A self-administered video-based Arabic language DA for chemotherapy decisions for metastatic colorectal cancer (mCRC) patients, given to patients after the oncology consultation. DA lists the 2 treatment options (with or without chemotherapy), presents the treatment benefit, response rates, and estimated survival rates. Information was presented using graphic illustrations and numeric estimates. The tool was intended to complement usual care. **Development** The DA was based on the Ottawa Decision Support Framework (ODSF). No report of a needs assessment amongst patients prior to development. **Comparator** Usual care alone	4 main oncology centres in Saudi Arabia: Metastatic colorectal cancer patients attending any one of the 4 main oncology centres. N = 92 Language: Arabic age 56.1 (SD 12.3) Education: Illiterate - 16 (17.4 %), Less than high school - 33 (35.9 %), High school - 16 (17.4 %), Diploma/College graduate/Postgraduate: 27 (29 %) Income: < 5000 SAR: 29 (31.5 %), 5000–10000 SAR: 41 (44.6 %), > 10000 SAR: 22 (23.9 %)	**Knowledge:** Self-developed Arabic questionnaire (score 0–8). (Measured at baseline, 1 month) **Anxiety** GAD 7 (general anxiety disorder - 7). (Measured at baseline, 1 month, 6 months)	No significant difference in understanding between DA and usual care groups at baseline (p = 0.86), and at 1 month (p = 0.45). Sociodemographic factors (> 50 % had low income and low education) had no impact on understanding. DA and usual care groups had mean reductions in anxiety (at 6 months); 2.27, *p* = 0.033 and 1.94, *p* = 0.045, respectively. NSD between arms.
Berry, DL 2013 RCT USA	The Personal Patient Profile-Prostate decision support for men with localized prostate cancer: A multi-centre randomized trial	**Description** The Personal Patient Profile Prostate (P3P) is a self-administered, web-based intervention that explores personal preferences, values, and concerns related to localised prostate cancer (LPC). It provides education in the form of text, graphs, and short video clips on medical facts and personally relevant factors to the prostate cancer decision, plus printed teaching sheets based on the patient’s priorities (Patients could “fill in the blank” text to use to prepare for the consultation). Coaching is provided in the form of personalised video vignettes (matched to race and age) to demonstrate patient-provider communication. Clinicians received a paper printout summarizing the P3P participants’ reports. Participants could access the P3P at home (with internet access) or in the clinic on touchscreen devices. **Development** Developed based on O’Connor’s Decision Support Framework (DSF). Patient needs assessment conducted prior to development. **Comparator** Control group: usual patient education resources for each clinic, typically books or pamphlets. *Both groups were offered links to reputable websites for prostate cancer*	6 institutions (urology, radiation oncology, or multi-disciplinary clinics in 4 geographically distinct areas. Men with newly diagnosed with T1 or T2, (localized) prostate cancer. All men required to read English or Spanish at ≥ grade 6 level. N = 494 Age: DA 63 (45–86). Control 62 (40–84) Minority/multiple race: 75 (15.2 %) Spanish/Hispanic/Latino: 15 (3 %)	**Decisional Conflict Scale (DCS)** [O′Connor] 16-item DCS consists of 5 items (uncertainty, informed, values clarity, support, effective decision). 5-point Likert scale Score: 0 (low conflict) - 100 (high conflict). (Measured at baseline, 1 month and 6 months). Spielberger State-Trait **Anxiety** Inventory (measured at baseline only)	Significantly reduced DCS scores in P3P arm over time for the 2 subscales of **uncertainty**, 3.61 units (95 % CI, 7.01–0.22) (P = 0.04), and **values clarity**, 3.57 units (95 % CI, 5.85–1.30) (P = 0.002). Inadequate information (informed subscale) was significantly associated with being older, non-White, and having less income, more trait anxiety and lower baseline information and support. Lack of values clarity was associated with less income, minimal pre-enrolment use of the Internet and inadequate support. Conflict related to perceived support significantly decreased as men were further from biopsy by number of weeks. The largest decrease between 1 and 2 weeks and associated with non-White race and baseline support scores.
Berry, DL 2018 RCT USA	Decision Support with the Personal Patient Profile-Prostate: A Multicentre Randomized Trial.	The Personal Patient Profile Prostate (P3P) is a self-administered internet-based DA for localised prostate cancer treatment (as described in Berry (2013)). The PtDA was revised to accommodate low-literacy users and tested with minority men during decision-making. (see Berry, DL, 2013, for details)	Urology and radiation clinics English and Spanish speaking men with newly diagnosed localized prostate cancer. N = 392 enrolled (276 analysed) Age: DA < 60 66 (33.3 %), > /= 60 132 (66.7 %), control < 60 59 (30.4 %), > /= 60 135 (69.6 %) Race: Black 113 (29 %), white (Hispanic) 12 (3 %), white (non-Hispanic) 240 (61 %) Education: > high school 310 (79 %), < /= high 77 (20 %)	**Decisional conflict DCS.** 10-item, low-literacy version. Score 0 (no conflict) – 40 (highest conflict)	In multivariable analysis, P3P intervention group reported a 5-unit (95 % CI, 0.59–9.40) lower DCS score (p = 0.03) at 1-month than the usual care group. In multivariable analysis: higher DCS at 1 month was associated with unemployment, lower income, having only 1 consultation, and high to intermediate risk cancer. Decision conflict varied significantly between sites.
Diefenbach, MA 2018 RCT USA	Examining the impact of a multimedia intervention on treatment decision-making among newly diagnosed prostate cancer patients: results from a nationwide RCT	**Description** “Healing Choices”. An online, video-based intervention. Participants were given access to the online program, instructions on how to access the program and received a CD-ROM version of the multimedia program. The program was sent to men after the telephone call. Not clear if this was before or after the consultation with the oncologist. The program provided information about cancer treatment options and side effects; addressed individual beliefs and expectations about cancer treatment and disease outcomes, provided emotional support through normalizing statements and a distress-lowering exercise; and modelled skills for decision-making to enhance self-efficacy in the consultation. Provided for patient determination of values and preferences regarding treatment and future QoL. **Development** Based on Self-regulation theory and social cognitive theory. A patient needs assessment was conducted before development. **Comparator** Usual personalised consultation with a CIS (cancer information service) specialist and print information materials	Cancer Information Services (CIS) Men with localised prostate cancer, who called the CIS for information, had access to a computer and were English speaking. N = 349 age 64.73 (SD 8.39) Education: High school or less 18.9%, some college: 26.6 %, college graduate/> 54.2 % Ethnicity: White 76.2 %, African American 16.9 %, other 2.6 % Income (USD) < 30000 21.2 %, 30000–59000 23.5 %, 60000–79000 17.8 %, > /= 80000 30.7 % Medical insurance 91.6 %	**Decisional Conflict** O’Connor DCS (5 subscales) Total scores 0 – 100 (highest decision conflict) (measured at 2 months) **Cancer related distress:** Intrusion subscale of the Impact of Event Scale (IES). Subscale score of ≥ 20 indicates clinically significant distress. (Measured at baseline and 2 months)	No significant difference in DCS score at 2 months: intervention group: mean 36.0 ± 12.03 vs comparison group mean 37.50 ± 12.60, p = 0.32). Significant effect on levels of perceived decisional support (intervention, M = 34.8, SD = 15.7; comparison, M = 38.3, SD = 16.1; p = 0.05). No significant difference between groups in in cancer-related distress at 2 months (p = 0.93) Race (tested as a two-group variable; White vs. African American) was the only significant moderator of decision support (p = 0.05). African American participants in the DA group reported greater decisional support compared to usual care. Patients with lower education levels benefitted more from the intervention with respect to perceived decisional support (p = 0.05).
Durand, M-A 2021 RCT USA	What matters most: randomized controlled trial of breast cancer surgery conversation aids across socioeconomic strata	**Description** Paper based in-consultation conversation DA. (2 options). 1. Option grid (OG) is a text-only 1-page DA providing information in simple language, on treatment options in a table form. 2. Picture Option Grid (POG) provides the same information with pictures and simpler language in 4 pages. Surgeons were trained in SDM and how to use their assigned intervention (POG or OG) before recruitment. **Development** No report of a framework supporting development. Patients included in usability, accessibility and acceptability testing. **Comparator** Usual care: Surgeons provided usual information.	4 NCI-designated cancer centres in urban and rural locations that provided care to a diverse population in terms of socio-economic status (SES), ethnic and racial populations. N = 616 Language: English 535 (86.9 %), Spanish 61 (9.9 %), other 13 (2 %) age mean 59.7 (SD 12.5) Education: > high school 421 (68.3 %) Race/Ethnicity: Black 96 (15.6 %), Hispanic 78 (12.7 %), White 394 (64 %), other 30 (4.9 %) SES: Lower 203 (33.0 %), higher 413 (67.1 %) Health literacy: low 276 (44.8 %), not low 333 (54.1 %)	**Decision quality** instrument with 19 items and 3 subscales (knowledge, concordance and decision process) adapted for persons with low socio-economic status (SES) **Shared decision making** CollaboRATE (a validated patient measure of **SDM**). Observer OPTION-5 to assess SDM in the consultation, by analysing the T1 audio recordings. Outcomes timeline: Baseline (T0), during the surgical visit (T1), immediately after the visit (T2), 1 week after surgery or within 2 weeks of the first postoperative visit (T3).	The POG and OG resulted in greater knowledge (T2 and T3) in comparison with usual care. NSD between the interventions (POG and OG) for knowledge. The POG resulted in higher self-reported and observed SDM in comparison with usual care. NSD between the interventions (POG vs OG) (estimate, 0.36; 95 % CI, 0.09–0.63; P = .01). **Author comments.** The POG reduced disparities in knowledge between patients of lower and higher SES compared with usual care. The POG improved more outcomes than the OG and had a positive impact on disadvantaged patients possibly due to inclusion of pictures.
Jalil N.B. 2020 RCT Malaysia (UMIC)	Effectiveness of Decision Aid in Men with Localized Prostate Cancer: a Multicentre Randomized Controlled Trial at Tertiary Referral Hospitals in an Asia Pacific Country	**Description** Modified version of a PDA based on studies of Western PDAs - a printed comprehensive treatment booklet with values clarification exercise. Available in three main languages (English, Malay, and Chinese). DA offered after diagnosis and treatment discussion visit, but prior to treatment decision visit. **Development** Input from local patients and doctors was sought. Cultural adaptations included evidence about complementary and alternative medicine and diet. **Comparator** Usual care	Six tertiary referral hospitals in four different states on the West Coast of Peninsular Malaysia. Patients with localised prostate cancer. N = 60 enrolled Age: ≥ 70 years: 26 (53.1 %) Ethnicity: Chinese = 37 (55 %) Education: secondary school or less = 25 (51 %) Income: ≤ RM 3999 (USD 975) = 30 (61%)	**Knowledge** A validated prostate cancer knowledge questionnaire adapted (Taylor et al. 2010). 23 items. (Max score = 23) **Decisional conflict DCS** (10 question version) Score 0 (no conflict) – 100 (extreme conflict)	Knowledge about prostate cancer: (baseline and post-intervention) Control group: 9.68 ± 4.58 vs 10.23 ± 3.52 (p 0.45) Intervention group: 9.19 ± 3.73 vs 11.26 ± 3.45 (p 0.02) Decision conflict: (baseline and post-intervention) Control group: 20.00 ± 24.59 vs 12.50 ± 23.54 (p 0.28) Intervention group: 26.48 ± 28.75 vs 12.41 ± 18.83 (p 0.01)
Jibaja-Weiss M. L. 2011 RCT USA	Entertainment education for breast cancer surgery decisions: A randomized trial among patients with low health literacy cancer patients early-stage disease.	**Description** A self-administered computerised, multimedia, interactive patient decision aid (CPtDA) entitled “A Patchwork of Life: One woman’s story for Making Breast Cancer Treatment Decisions”, combining soap opera episodes with interactive learning modules for breast cancer surgery options (breast conserving surgery (BCS) vs mastectomy). Targeted at multi-ethnic, low-literacy women. DA was available online or as a CD-ROM. The DA was accessed after the diagnosis, prior to decision visit. Printouts were generated for the women and put into their files. A case manager was available to assist women with accessing the DA on the computer and to provide emotional support. **Development** DA developed based on edutainment concepts and theory and the ODSF. **Comparator** Control group received usual care (UC), printed information about breast cancer treatment and material from national cancer institute and the American Cancer Society.	Breast pathology clinics in two public hospitals in the Harris County Hospital District. Women with early-stage breast cancer eligible for breast conserving surgery. Patients **do not have medical insurance, with many having a high school education or less.** N = 138 (completed = 76) Age 51. years (SD 10.9) Language: English 89 (65 %), Spanish 49 (35 %) Ethnicity: White 22 (15 %), African American 52 (38 %), Hispanic 62 (45 %), Asian American 2 (2 %)	**Knowledge** Breast cancer and treatment **knowledge** using an adapted questionnaire with 16 questions. (Measured pre-decision, pre-surgery and 1 year later). **Decisional conflict DCS** (10-item low-literacy version) (Measured pre-decision, pre-surgery and 1 year later). Surgical **treatment preference** (BCS, modified radical mastectomy, unsure), at pre-surgery visit.	Pre-surgery, significant increase in knowledge for CPtDA group and none in control (p < 0.001). Decreased DCS for all scales over the periods of assessment. At pre-surgery assessment, DCS was significantly lower for feeling informed (p = 0.007) and marginally for clarity of personal values (p = 0.053) in CPtDA group. CPtDA program patients were less likely to prefer breast-conserving surgery (40.5 % vs. 50.0 %), and more likely to prefer modified radical mastectomy (59.5 % vs. 39.5 %, P = 0.018), compared to UC patients. No CPtDA patients were unsure about their surgical preference compared to 10.5 % of controls.
Joshi, S 2023 RCT India	Effectiveness of a Decision Aid Plus Standard Care in Surgical Management Among Patients With Early Breast Cancer. A Randomized Clinical Trial	**Description** The Navya–Patient Preference Tool (Navya-PPT), is a self-administered, online, adaptive, conjoint analysis-based decision aid and patient preference assessment tool. The adaptive, conjoint analysis-based module represents trade-offs between 3 main attributes: ability to preserve cosmesis with breast-conserving surgery, adverse effects, and additional cost of mandatory radiation following breast-conserving surgery. Evidence supporting each treatment option is communicated in a pictorial format. Patients enter their preferences for each attribute on a Likert scale from 1 to 9, which determines the patient’s values for each of the trade-offs represented by survey questions. The Navya-PPT was translated from English into Hindi and Marathi. **Development** Jointly developed by researchers at Tata Memorial Centre, Navya Network, and Harvard University. **Comparator** Patients received clinician counselling on surgical options as standard care.	Outpatient clinic at the Tata Memorial Centre, a high-volume tertiary care cancer centre in Mumbai. Women with early breast cancer (cT1–2/cN0). N = 255 enrolled, 10 excluded after randomisation. Age: median (range) was 48 (23–76) years. SES: 144 participants (58.6 %) of lower or middle socioeconomic status Education: 114 (46.4 %) had not completed college. API-DM (Autonomy Preference Index—Decision-Making): Mean (SD) 4.38 (0.78) TEGR (Traditional-Egalitarian Gender Role): Mean (SD) 3.85 (0.87) CG (Caregiving): Mean (SD) 4.42 (0.81)	**Decisional Conflict DCS** (16-item questionnaire, Likert scale from 1 (strongly agree) to 5 (strongly disagree) - higher mean scores indicate higher decisional conflict. API-DM (Autonomy Preference Index-Decision-Making). 6 items, 5-point Likert scale (1 = ‘Strongly disagree’ and 5 = ‘Strongly agree) mean higher score = less desire for autonomy.	Significant decrease in DCS score in the solo group (patient alone) vs the control group (1.34 vs 1.66; 95% CI, 0.12–0.52; P < .001) and in the joint group (patient + caregiver) vs the control group (1.31 vs 1.66; 95% CI, 0.15–0.55; P < .001) Age, education and socioeconomic factors did not significantly impact on the DCS. Decision control preference, traditional-egalitarian gender roles and caregiving roles had no significant impact on the change in DCS in the intervention groups. **Author comment:** “Women who are less autonomous, more traditional, and more responsible for caregiving functions in the family likely have inherent disparities in access to shared decision-making… the use of Navya-PPT can reduce DCS score in women regardless of decreased autonomy, adherence to more traditional gender norms, or higher caregiving burden.”
Li K.D 2021 Analytical cross-sectional USA	Differences in Implementation Outcomes of a Shared Decision-Making Program for Men with Prostate Cancer between an Academic Medical Center and County Health Care System (LAC-DHS).	**Description** WiserCare DA for prostate cancer offered before the clinic visit. A validated DA available in English and Spanish, that integrates high quality evidence with patient clinical data and preferences. DA educated patients about different prostate cancer treatments and explored personal preferences and treatment goals using conjoint analysis. Internet based; self-administered DA accessed online from an emailed secure link. Patients without access at home were assisted by research staff to complete the DA in the clinic before their appointment with the doctor. Patients received an electronic report of their clinical details, personal values and treatment options. The one-page report was emailed to the doctor prior to the appointment. **Development** Not reported **Comparator** None	Academic Medical Centre and County Health Care System (LAC-DHS). Men with newly diagnosed non-metastatic prostate cancer. Able to read English or Spanish. N = 277 Age: Total 63 (IQR 58, 68) Race/ethnicity: White: total 118 (46 %) Black: total 27 (11 %) Asian total 22 (9 %) Hispanic/Latino total 26 (10 %) Other total 61 (26.9 %) The county DHS facilities had a higher percentage of non-White men than at the academic centre (83% v. 45 %, P = 0.001), and younger patients (61 years vs 64 years, p = 0.001)	**Decision control preference** Control preferences Scale **Decisional Conflict DCS** (16 item, 5-point likert scale with 5 domains). Score 0–100 (highest decision conflict) **Shared Decision-Making** Questionnaire (SDM-Q-9). Score 0–45 (Highest SDM)	Most patients at both sites preferred shared or active **decision role:** (academic centre 98 % v. county 90 %, P = 0.25). The median total **decisional conflict** after using the DA, was 7 (interquartile range [IQR] 0, 25) at the academic centre and 13.3 (IQR 3.9, 29.7) at county. NSD between sites in total DC or in the 5 subscales. **SDM** median scores of 36 (IQR 30, 44) at the academic centre vs 42 (IQR 32, 45) at the county. NSD between 2 sites (P = 0.39).
Negarandeh R. 2023 RCT Iran (LMIC)	Evaluation of a Question Prompt List on Treatment Decision-making Outcomes in women Following Surgery for Breast Cancer: A Randomized Controlled Trial	**Description** Question prompt list (QPL) self-administered prior to the consultation, with 14 questions for each breast cancer treatment in 3 domains: (1) information about the malignancy; (2) treatment options; (3) choices for follow-up after treatment. Answers to the QPL questions were provided in the subsequent consultation in the form of face-to-face contacts, telephone, or through the WhatsApp social network tool. The intervention occurred prior to the final treatment decision consultation. **Development** The QPL was developed using 3 sources of information: (1) existing evidence from other chemotherapy and radiotherapy-related QPLs; (2) expertise from the healthcare team in the radiotherapy-oncology department; and (3) interviews conducted with patients before the study. The QPL was tested for validity, accuracy and comprehensiveness. **Comparator** Usual care (received information about their respective treatments).	Comprehensive cancer centre in Tehran. Post-surgical breast cancer patients for follow-up treatment planning. Ability to read and write in Persian. N = 50 Age: mean (SD) = 50,04 (11.36) Education: less than high school 28 (56 %)	**Shared decision-making** Shared Decision-Making Questionnaire - SDM-Q-9 (6-point likert scale). Score 0–45 (Highest SDM) **Decision control preference** Control preferences Scale	SDM-Q-9: Control 40 (SD 7.5) vs intervention 43 (SD 5.75) (p 0.17) CPS: (pre- vs post-intervention – for preferred passive role) Control group: (23 (92 %) vs 19 (86.4 %) vs Intervention group: (24 (96 %) vs 23 (98.8 %) (p 0.46)
Tilburt JC 2022 RCT USA	Decision Aids for Localized Prostate Cancer in Diverse Minority Men: Primary Outcome Results from a Multi-Centred Cancer Care Delivery Trial (Alliance A191402CD)	**Description** **1. Prostate Cancer Choice Decision Aid** A within-visit electronic “light text” DA, used in the consultation by clinicians to discuss treatment choices with patients. The DA assessed quality of life and provided individualized estimates of prostate cancer risk and life expectancy. Patients also rated the importance of oncologic outcomes and quality of life. A summary page was provided after the consultation. **2. Knowing Your Options Decision Aid** A pre-visit “text-heavy” DA provided men with localized prostate cancer detailed information about their cancer and treatment options using video, images and risks communicated visually. Provides values clarification regarding treatment options and provides a summary to be printed. Designed to be used prior to or after the treatment discussion consultation. Participating physicians were orientated by video and were offered on-site training in the tool as needed. **Development** Both DAs developed according to the international standards for decision aid development. Focus groups with patients and doctors informed development. **Comparator** Usual care	15 urology practices affiliated with NCI-funded NCORP (NCI Community Oncology Research Program) sites that received funding to conduct cancer care delivery research (CCDR). Clinically localized (T1–3) prostate cancer. English literate or have access to translation/interpreter assistance. N = 155 Age: mean (SD) = 63.5 (7.7) Black or African American = 85 (53.8 %)	**Knowledge** measured after the index treatment discussion consultation. Investigator designed 12-item yes/no questionnaire. Number of correct responses were converted to a proportion. Data reported as: mean proportion (SD).	Knowledge: mean proportion (SD) Pre-visit DA only: 0.65 (0.164) Within-visit DA only: 0.58 (0.167) Pre-visit and within-visit DAs: 0.69 (0.165) Neither DA: 0.56 (0.232) Mean difference in post-visit knowledge was 0.094 (97.5 % CI: −0.055, 0.242) and 0.002 (97.5 % CI: −0.147, 0.150) for the pre-visit decision aid and for the within-visit decision aid, respectively. Neither decision aid intervention effect achieved statistical significance at the pre-specified 2.5 % level (*P* = 0.132 and 0.977, respectively). Analysis within the racial/ethnic subgroup (other than non-Hispanic white) also showed NSD between interventions or control.
Wang S 2022 Quasi-experimental China (UMIC)	Effects of a smartphone application named “Shared Decision-Making Assistant” for informed patients with primary liver cancer in decision-making in China: a quasi-experimental study	**Description** A smartphone application: “SDM Assistant” included two core parts: “PLC Treatment Knowledge Centre” and “Decision Aids Path”. The “PLC Treatment Knowledge Centre” includes the epidemiology of PLC and clarification of the 12 PLC treatment schemes, each of which includes information from indications to treatments with advantages and disadvantages, recurrence rates and health education after discharge. The language is at a sixth grade reading level. The modules of the “SDM Assistant” are presented as video, audio, text, 3D image, treatment scheme effect comparison diagram, etc. It includes a 5-step process with an initial and final consultation with the doctor. The app was self-administered with nurses in the ward assisted with installing the app and explaining how to use it. **Development** Based on the Ottawa Decision Support Framework. **Comparator** Usual care	Eastern Hepatobiliary Surgery Hospital, Naval Medical University, Shanghai, China. Primary liver cancer patients admitted to 2 wards of the same department. N = 190 Education: < high school 26 (14.4%), high school 129 (67.9 %) Tertiary 35 (19.5 %) Ave monthly income: ≤ 3000 yuan (US$ 435) 58 (63.4 %)	**Knowledge** Pre- and post-interventions using 17 items in reference to Breast Cancer Knowledge Scale. Knowledge domains included liver function, PLC risk factors, PLC screening, and PLC symptoms. Response options for each item were “true”, “false”, or “I don’t know”. A maximum total knowledge score of 17 (1 point for each correct item). **Decisional Conflict Modified Chinese version of DCS** [O′Connor] 16-item DCS. 3 subscales: (1) uncertainty about choosing among different options, (2) modifiable factors that cause this uncertainty, and (3) perceived effectiveness of the choice. Total scores range: 0 (no decision conflict) - 100 (highest-level in decision conflict). Measured pre- and post-intervention.	**Knowledge post intervention:** Intervention group 12.72 (± 2.13) vs control group 14.52 (± 1.91), (p 0.001) **Decision conflict post-intervention:** Intervention group: 16.89 ± 8.80 vs. control group: 26.75 ± 9.79, (*P* 0.001)
Wyld L 2021 (Multi-centre cluster RCT). England and Wales	Bridging the age gap in breast cancer: cluster randomized trial of two decision support interventions for older women with operable breast cancer on quality of life, survival, decision quality, and treatment choices.	**Description** Decision support interventions (DESI) for older women with breast cancer. DESIs include 1) a booklet with information and values clarifying exercises for each treatment decision, 2) brief decision aids composing of FAQs with answers to be used in the consultation, 3) a validated online algorithm (Age Gap Decision Tool based on UK cancer registry survival data) used by the doctor in the consultation. The algorithm produced personalized survival outcomes for women aged 70 years or above according to fitness, frailty, stage, treatment choice, and disease biology. Staff at intervention sites received training in the use of the DESIs to support SDM. Two key treatment choices: 1) surgery plus adjuvant endocrine therapy versus primary endocrine therapy, and 2) surgery plus adjuvant chemotherapy versus no chemotherapy. **Development** The Coping in Deliberation (CODE) framework formed the theoretical basis for the DESI. The DESIs used the preferred informational content, format, terminology, and media for women over 70 years of age. **Comparator** Usual decision-making practices.	Women recruited from 46 breast units in England and Wales, who were 70 years or older with primary operable breast cancer, and able to read English. Intervention sites offered the DESI to all women regardless of enrolment status. 46 clusters (21 intervention, 25 control) n = 1339 (670 intervention, 669 control) **age 77** (IQR 70–96, range 69–102)	**Knowledge:** 8 item bespoke questionnaire (Score 0–8), measured before and 6 weeks after decision-making. **Shared decision making** CollaboRATE (3 item **SDM** measure scored 0 (no effort) −9 = (every effort to promote SDM)) transformed into a scale of 0–100 **Anxiety** Spielberger Short State-Trait Anxiety Inventory (6 items), measured before treatment, 6 weeks and 6 months after decision-making.	Greater knowledge by patients in the intervention arm (median scores 5/8 versus 3/8; P < 0.001) and more participants in the intervention arm stated that they knew the available options (94 % vs 74 %; P 0.003) and associated advantages (91 % vs 76 %; P-0.054). Patients reported a high quality of shared decision-making, with a median score of 100 in both arms (P 0.729). No difference in STAI scores at 6 weeks or 6 months.
Yen RW 2020 RCT USA	Text-only and picture conversation aids both supported shared decision making for breast cancer surgery: Analysis from a cluster randomized trial.	**Description** An in-consultation tool used by clinicians and patients. Presented the treatment options, with balanced information and pros and cons; and structured the conversation, encouraging patient-centred dialogue. 1. Option grid (OG) is a text-only 1-page DA providing information in simple language, on treatment options in a table form. 2. Picture Option Grid (POG) provides the same information with pictures and simpler language in 4 pages to facilitate understanding of patients with lower health literacy. Frequently Asked Questions providing evidence-based information on two surgical options for breast cancer. Surgeons were trained in the use of the tools. **Development** The DAs were derived from an evidence-based table comparing treatment options for breast cancer (http://www.optiongrid.org) **Comparator** Usual care (UC)	Women with localised breast cancer deciding between breast-conserving surgery plus radiation vs mastectomy, recruited from 4 NCI-designated cancer centres. n = 311 age 60.5 (SD 12.2) Language: English 283 (91.0 %), Spanish 24 (7.7 %), other 4 (1.3 %) Race/ethnicity Black, non-Hispanic 35 (11.3 %), Hispanic 30 (9.6 %), white non-Hispanic 229 (73,5 %), other 11 (3.5 %) Education: Less than college 201 (64.4 %), college or higher 110 (35.4 %) Socio-economic status (SES) Low 104 (33.4 %), high 207 (66.6 %) Health literacy inadequate 141 (45.3 %) adequate 168 (54.0 %) Insurance private 220 (70.7 %), public/uninsured 91 (29.3 %)	**Shared decision making Patient-reported SDM** using CollaboRATE, Scores above 40 corresponds to at least baseline ability in SDM. **Observed SDM** from recordings of consultations using observer - OPTION-5 score assessment.	Top scores on CollaboRATE: OG arm (81.6 %), POG (80.0 %), UC (56.4 %), p < 0.001. No significant difference in scores between OG and POG. (p > 0.05). The mean OPTION-5 scores: OG arm - 73.0 (SD = 14.2), POG arm 56.3 (SD = 21.9), UC arm - 41.0 (SD = 27.5), p < 0.0001 (ANOVA). Mixed-effects multilevel regression model adjusted for surgeon, clinic, and site clusters: OG was associated with a 32.6 (95 % CI 14.8, 50.4) point increase in OPTION-5 score compared to UC (p < 0.001). POG was associated with a 23.7 (95 % CI 7.4,40.0) point increase in OPTION-5 score (p = 0.004) compared to UC. The crude mean OPTION-5 scores for patients with lower SES were 43.1 and for patients with higher SES were 56.8 (p < 0.0001). Crude mean OPTION-5 scores for patients of inadequate health literacy were 50.9 and for patients of adequate health literacy were 52.9 p = 0.50). There were significant differences in scores when stratifying by arm for SES but not for health literacy.

**Table 4:** Synthesised findings and major themes from the qualitative articles.

Synthesised finding 1	Themes
Patients differed in terms of their information needs and accessing sources of information, desired content and format.	1. Patient information need 2. Patient information gathering 3. Patients’ preferred information format and presentation
Synthesised finding 2	Themes
Decision making is a process that starts before the treatment decision consultation, requires effort from the patient to consider options under stressful circumstances, often making choices that are not only based on clinical information.	1. Process and timing of decision making 2. Work of deliberation on options/choices 3. Reasons for treatment decision choices
Synthesised finding 3	Themes
Decision making consultations are affected by the style of clinician and patient interaction impacting on decision control, where patients sometimes adopted roles that they did not prefer, and preferred format of decision supports varied.	1. Clinician patient interactions 2. Preferred or perceived passive patient decision role 3. Preferred or perceived active decision role 4. Consultation decision support
Synthesised finding 4	Themes
Patients experienced strong emotions following a cancer diagnosis that impacted on their ability to make decisions. Social, cultural and language factors influenced decision making, indicating the need for an enhanced psychosocial approach to decision support.	1. Emotional/Social impact of cancer and treatment decision making 2. Family, social, cultural influences and language barriers in decision making 3. Strategies for socio-emotional support

**Table 5:** Integrated synthesis approach for qualitative and quantitative studies.

Qualitative synthesis	Quantitative synthesis
**Synthesised finding 1**	**Knowledge outcomes**
Patients differed in terms of their information needs and accessing sources of information, desired content and format.	Cancer and/or treatments
**Synthesised finding 2**	**Decision conflict outcomes**
Decision making is a process that starts before the treatment decision consultation, requires effort from the patient to consider options under stressful circumstances, often making choices that are not only based on clinical information.	
	Uncertainty Informed Values clarity Support Effective decision
**Synthesised finding 3**	**Shared decision making**
Decision making consultations are affected by the quality of clinician and patient interaction impacting on decision control, where patients sometimes adopted roles that they did not prefer, and preferred format of decision supports varied.	
	Observed Self-reported
**Synthesised finding 4**	**Anxiety/cancer related distress**
Patients experienced strong emotions following a cancer diagnosis that impacted on their ability to make decisions. Social, cultural and language factors influenced decision making, indicating the need for an enhanced psychosocial approach to decision support.	

**Table 6:** Integration of the qualitative needs finding and PtDA content and effectiveness in addressing the needs.

	AlSagheir	Berry 2013 and 2018*	Diefenbach		Durand and Yen**		Jibaja-Weiss	Li		Tilburt	Wyld	Joshi	Jalil	Negarandeh	Wang	1st Author, Quantitative articles
**PtDA**	Arabic DA	P3P	Healing choices		OG	POG	CPtDA	Wiser-CARE	Prostate cancer choice	Knowing your options	DESI	Navya-PPT	PDA	QPL	SDM Assistant	
**Qualitative findings**	**HIC**											**LMIC**				**Quantitative PtDA content and effectiveness**
**Qualitative INFORMATION needs findings addressed by PtDAs**																
Information content need	√	√	√		√	√	√	√	√	√	√	√	√		√	Provided cancer &/or treatment information
		√	√				√	√	√	√						Individually tailored information
Information format need		√	√				√	√	√	√	√	√			√	Online (web-based)
					√	√					√		√	√		Paper-based (print)
	√	√	√				√			√					√	Video
	√	√				√				√	√	√			√	Pictures/ graphs
Information gathering	√	√	√				√	√		√	√	√	√	√	√	Self-administered
							√	√								Self-administered with staff help
	**DECISION AID EFFECTIVENESS IN ADDRESSING THE INOFRMATION NEEDS**															
**Finding 1**	**NSD**				↑	↑	↑		**NSD**	**NSD**	↑		↑		↑	**Knowledge outcomes**
**Qualitative DECISION SUPPORT needs findings addressed by PTDAs**																
Decision making work		√	√				√								√	Coaching (Deliberation steps)
Deliberation on		√	√				√	√	√	√	√	√	√		√	Values clarification
options		√					√	√	√	√						Summary page patient/doctor
Process and timing		√					√	√		√	√	√	√		√	Before treatment decision visit
	**DECISION AID EFFECTIVENESS IN ADDRESSING THE DECISION SUPPORT NEED**															
**Finding 2**		↓	↓				↓	↓				↓	↓		↓	**Decision conflict outcomes**
**Qualitative DECISION-MAKING PARTICIPATION needs findings addressed by PTDAs**																
Clinician-patient interaction					√		√		√		√			√	**√**	In-visit (consultation) use
	**DECISION AID EFFECTIVENESS IN ADDRESSING THE DECISION-MAKING PARTICIPATION NEED**															
**Finding 3**					↑	↑		↑			**NSD**			**NSD**		**Shared decision-making outcomes**
**Qualitative PSYCHOSOCIAL SUPPORT needs findings addressed by PtDAs**																
Emotional distress			√				√									Emotional support
	**DECISION AID EFFECTIVENESS IN ADDRESSING PSYCHOSOCIAL SUPPORT NEEDS**															
**Finding 4**	**NSD**		**NSD**								**NSD**					**Anxiety/Distress outcomes**

## Data Availability

The data underlying this article are available in the article and the online supplementary material.

## Appendix A. Supporting information

Supplementary data associated with this article can be found in the online version at doi:10.1016/j.pec.2026.109491.
