# Supplementary material for: A mixed methods systematic review of cancer treatment decision-making in vulnerable populations
Source: Patient Educ Couns. Author manuscript; Available in PMC 2026 Jul 30. (PMC13419551; doi:10.1016/j.pec.2026.109491)
Supplement: 1 [file NIHMS2189791-supplement-1.docx]

**Supplementary Table A.1. Search strategy**

| **Pubmed** | |
| --- | --- |
| #1 | (neoplasms [MeSH Terms]) OR (cancer*[Title/Abstract] OR oncolog*[Title/Abstract] OR tumor*[Title/Abstract] OR tumour*[Title/Abstract] OR malignanc*[Title/Abstract] OR neoplasm*[Title/Abstract]) |
| #2 | (Decision making, shared [MeSH Terms]) OR ("Informed Consent" AND "Health Care Quality, Access, and Evaluation"[MeSH Terms]) |
| #3 | "Shared decision making"[Title/Abstract] OR "Informed decision making"[Title/Abstract] OR "Patient decision making"[Title/Abstract] OR "patient clinician communication"[Title/Abstract] OR "joint decision making"[Title/Abstract] OR "patient engagement"[Title/Abstract] OR "patient involvement"[Title/Abstract] OR "patient participation"[Title/Abstract] |
| #4 | "patient engagement"[Other Term] OR "patient involvement"[Other Term] OR "patient participation"[Other Term] |
| #5 | #2 AND #3 AND #4 |
| #6 | Decision support techniques OR Decision Support Systems [MeSH Terms] |
| #7 | "decision support*"[Title/Abstract] OR "decision aid*"[Title/Abstract] OR "decision tool*"[Title/Abstract] OR "decision support system*"[Title/Abstract] OR "decision support technique*"[Title/Abstract] |
| #8 | #6 AND #7 |
| #9 | (Patient Preference OR Patient Satisfaction MeSH Terms]) OR (Patient experience*[Title/Abstract] OR patient perception*[Title/Abstract] OR patient view*[Title/Abstract] OR patient preference*[Title/Abstract] OR patient satisfaction [Title/Abstract]) |
| #10 | (Patient experience*[Title/Abstract] OR patient perception*[Title/Abstract] OR patient view*[Title/Abstract]) OR (Patient experience*[Other Term] OR patient perception*[Other Term] OR patient view*[Other Term]) |
| #11 | ((Patient Preference OR Patient Satisfaction [MeSH Terms]) OR ("patient preference*"[Title/Abstract] OR "patient satisfaction"[Title/Abstract])) OR ((Patient experience*[Title/Abstract] OR patient perception*[Title/Abstract] OR patient view*[Title/Abstract]) OR (Patient experience*[Other Term] OR patient perception*[Other Term] OR patient view*[Other Term])) |
| #12 | #1 AND #5 AND #8 |
| #13 | #1 AND #5 AND #8 AND #11 |
| #14 | (("vulnerable populations"[MeSH Terms]) OR (vulnerable p*[Title/Abstract])) |
| #15 | #12 AND #14 |
| #16 | (((("Developing Countries"[Mesh]) OR ("low to middle income countr*"[Title/Abstract])) OR ("middle income countr*"[Title/Abstract])) OR ("low income countr*"[Title/Abstract])) |
| #17 | #12 AND #16 |
| **Medline** | |
| #1 | ((MW neoplasm) OR (TI "neoplasm*" OR AB "neoplasm*”) OR (TI "cancer*" OR AB "cancer*”) OR (TI "oncolog*" OR AB "oncolog*”) OR (TI "tumor*" OR AB "tumor*) OR (TI "tumour*" OR AB "tumour*") OR (TI "malignanc*" OR AB "malignanc*" )) |
| #2 | ((MW Decision making, shared) OR ( TI “Shared decision making” OR AB “Shared decision making” ) OR ( TI “Informed decision making” OR AB “Informed decision making” ) OR ( TI “Patient decision making” OR AB “Patient decision making” ) OR (TI “patient clinician communication” OR AB “patient clinician communication” ) OR ( TI “informed consent" OR AB “informed consent" ) ) OR ( ( TI “patient engagement” OR AB “patient engagement” ) OR ( TI “patient involvement” OR AB “patient involvement” ) OR ( TI “patient participation” OR AB “patient participation” ) |
| #3 | ((MW decision support techniques OR MW decision support systems) OR (( TI “decision support*” OR AB “decision support*”) OR ( TI "decision aid*” OR AB "decision aid*”) OR ( TI “decision tool*” OR AB “decision tool*”) OR ( TI “decision support system*” OR AB “decision support system*”) OR ( TI “decision support technique*” OR AB “decision support technique*”)) |
| #4 | ((MW patient preference OR MW patient participation) OR ((TI "patient preference*" OR AB "patient preference*”) OR (TI "patient participation" OR AB "patient participation") OR (TI “Patient experience*” OR AB “Patient experience*”) OR (TI “patient perception*” OR AB “patient perception*”) OR TI “patient view*” OR AB “patient view*”)) |
| #5 | #1 AND #2 AND #3 |
| #6 | #5 AND #4 |
| #7 | ((MM "Vulnerable Populations") OR (MM" Health Disparate Minority and Vulnerable Populations")) OR TI ("Vulnerable Populations "OR "vulnerable person*"OR "minority populations") OR AB (“Vulnerable Populations" OR" vulnerable person*" OR “minority populations”) |
| #8 | ((MM "Developing Countries") OR TI ("developing countr*" OR "low income countr*" OR "middle income countr*”) OR AB (“developing countr*" OR "low income countr*" OR "middle income countr*" )) |
| #9 | #5 AND #7 |
| #10 | #5 AND #8 |
| **CINAHL** | |
| #1 | ( MW "Neoplasms" OR MH "Oncology+" ) OR ( (MH "Radiation Oncology") OR (MH "Cancer Patients") OR (MH "Oncology Surgery") OR (MH "Oncologic Care") OR (MH "Oncology") ) OR ( ( TI oncolog* OR AB oncolog* ) OR ( TI cancer* OR AB cancer* ) OR ( TI malignanc* OR AB malignanc* ) OR (TI tumor* OR AB tumor* ) OR ( TI tumour* OR AB tumour* ) ) OR ( ( TI "cancer patient" OR AB "cancer patient" ) OR ( TI "oncology patient" OR AB "oncology patient" ) OR ( TI "patient with cancer" OR AB "patient with cancer")) |
| #2 | ( MH "Decision Making, Shared" OR MH "Decision Making, Patient" ) OR ( TI "shared decision making" OR AB "shared decision making" ) OR ( TI "informed decision making" OR AB "informed decision making" ) OR ( TI "patient decision making" OR AB "patient decision making" ) OR (TI “patient clinician communication” OR AB “patient clinician communication” ) OR ( TI “joint decision making” OR AB “joint decision making” ) OR ( TI “patient clinician communication” OR AB “patient clinician communication)) |
| #3 | (MH "decision support systems, clinical" OR MH "decision support techniques" OR (MH "decision-making support (Iowa NIC)" ) OR ( TI “decision support*” OR AB “decision support*” ) OR ( TI “decision intervention*” OR AB “decision intervention*” ) OR ( TI “decision tool*” OR AB “decision tool*” ) OR (TI “decision aid*” OR AB “decision aid*” ) |
| #4 | (MH "patient preference" OR MH "patient satisfaction" ) OR ( TI "patient perception*" OR AB "patient perception*" ) OR ( TI "patient attitude*" OR AB "patient attitude*" ) OR ( TI "patient opinion*" OR AB "patient opinion*" ) OR ( TI "patient experience*" OR AB "patient experience*" ) OR ( TI "patient perspective*" OR AB "patient perspective*" ) OR ( TI "patient view*" OR AB "patient view*" ) OR (TI "patient feeling*" OR AB "patient feeling*" ) OR ( TI "patient thought*"OR AB "patient thought) |
| #5 | #1 AND #2 AND #3 |
| #6 | #5 AND #4 |
| #7 | (MH developing countries OR developing nations OR low income countries ) OR (MH developing countries OR developing nations OR low income countries ) OR TI (“low to middle income countr*" OR "middle income countr*"OR "low income countr*") OR AB (“low to middle income countr*" OR "middle income countr*" OR "low income countr*") |
| #18 | (MW "vulnerable populations" OR MH "vulnerable populations") OR (TI “vulnerable population*" OR "disadvantaged population*" OR "under-served population*”) OR AB (“vulnerable population*" OR "disadvantaged population*" OR "under-served population*”) |
| #19 | #5 AND #7 |
| #10 | #5 AND #8 |
| **PsychInfo** | |
| #1 | (MA neoplasms) OR (TI "neoplasm*" OR AB "neoplasm*”) OR ((TI "cancer*" OR AB "cancer*”) OR (TI "oncolog*" OR AB "oncolog*”) OR TI "tumor*" OR AB "tumor*”) OR (TI "tumour*" OR AB "tumour*") OR (TI "malignanc*" OR AB "malignanc*")) OR (KW "cancer*" OR "oncolog*" OR "tumor*" OR "tumour*" OR "malignanc*" OR "neoplasm*") |
| #2 | (MA "decision support techniques" OR "decision support systems") OR (TI “decision support*” OR AB “decision support*”) OR (TI "decision aid*” OR AB "decision aid*”) OR (TI “decision tool*” OR AB “decision tool*”) OR (TI “decision support system*” OR AB “decision support system*”) OR (TI “decision support technique*” OR AB “decision support technique*”) OR (KW “decision support*” OR "decision aid*” OR “decision tool*” OR “decision support system*”) |
| #3 | (MA decision making, shared) OR ((TI “Shared decision making” OR AB “Shared decision making”) OR (TI “Informed decision making” OR AB “Informed decision making”) OR (TI “Patient decision making” OR AB“Patient decision making”) OR (TI “patient clinician communication” OR AB “patient clinician communication”) OR (TI “informed consent" OR AB “informed consent")) OR (KW “Shared decision making” OR “Informed decision making” OR “Patient decision making” OR “patient clinician communication” OR “informed consent”) |
| #4 | (MA "patient preference" OR "patient satisfaction") OR ((TI "patient preference*" OR AB "patient preference*") OR (TI "patient participation" OR AB "patient participation") OR (TI “Patient experience*” OR AB “Patient experience*”) OR (TI “patient perception*” OR AB “patient perception*”) OR (TI “patient view*” OR AB “patient view*”)) OR( KW “patient experience*” OR “patient perception*” OR “patient view*” OR “patient preference*” OR “patient satisfaction”) |
| #5 | #1 AND #2 AND #13 |
| #6 | #5 AND #4 |
| #7 | (MA vulnerable populations or groups or people) OR TI (vulnerable populations or vulnerable or vulnerability or minority groups or 'hard to reach populations’) OR AB (vulnerable populations or vulnerable or vulnerability or minority groups or 'hard to reach populations') OR KW (vulnerable populations or vulnerable or vulnerability or minority groups or 'hard to reach populations') |
| #8 | MA (developing countries or developing nations or third world or low income countries) OR TI (“developing countr* OR "low income countr*" OR "middle income countr*") OR AB ("developing countr* OR "low income countr*" OR "middle income countr*") OR KW ("developing countr* OR "low income countr*" OR "middle income countr*") |
| #9 | #5 AND #7 |
| #10 | #5 AND #8 |
| **Cochrane Library** | |
| #1 | [Neoplasms] MeSH OR (neoplasm*):ti,ab,kw OR (cancer*):ti,ab,kw OR (tumo?r):ti,ab,kw OR (malignanc*):ti,ab,kw OR (oncolog*):ti,ab,kw |
| #2 | [Decision Making, Shared] MeSH OR [Informed Consent] MeSH OR ("shared decision making"):ti,ab,kw OR ("informed decision making"):ti,ab,kw OR ("patient decision making"):ti,ab,kw OR ("joint decision making"):ti,ab,kw OR ("patient clinician communication"):ti,ab,kw |
| #3 | [Decision Support Techniques] MeSH OR [Decision Support Systems, Clinical] MeSH OR ("decision support* "):ti,ab,kw OR ("decision aid*"):ti,ab,kw OR ("decision tool*"):ti,ab,kw OR ("decision support system*"):ti,ab,kw OR ("decision support technique*"):ti,ab,kw |
| #4 | [Patient Preference] MeSH OR [Patient Satisfaction] MeSH OR ("Patient experience*"):ti,ab,kw OR ("patient perception*"):ti,ab,kw OR ("patient view*"):ti,ab,kw OR ("patient preference*"):ti,ab,kw OR ("patient satisfaction"):ti,ab,kw OR ("patient engagement"):ti,ab,kw OR ("patient involvement"):ti,ab,kw |
| #5 | #1 AND #2 AND #3 |
| #6 | #5 AND #4 |
| #7 | [Vulnerable populations] MEsH OR (“vulnerable person*”):ti,ab,kw OR (disadvantaged person*):ti,ab,kw OR (“underserved person*”):ti,ab,kw OR (“minorit*” person*”):ti,ab,kw |
| #8 | [Developing countries] MeSH OR (“low income countr*”):ti,ab,kw Or (“middle income countr*”):ti,ab,kw |
| #9 | #5 AND #7 |
| #10 | #5 AND #6 |
| **Global Health** | |
| #1 | ((TI neoplasm* OR oncolog* OR cancer* OR tumor* OR tumour*OR malignanc*) OR (AB neoplasm* OR oncolog* OR cancer* OR tumor* OR tumour* OR malignanc*) OR (KW neoplasm* OR oncolog* OR cancer* OR tumor* OR tumour*OR malignanc*)) |
| #2 | ((TI "shared decision making" OR "informed decision making" OR “patient decision making" OR "patient clinician communication" OR "informed consent") OR (AB "shared decision making" OR "informed decision making" OR “patient decision making" OR "patient clinician communication" OR "informed consent") OR ((TI "patient engagement" OR "patient involvement" OR "patient participation") OR (AB "patient engagement" OR "patient involvement" OR "patient participation") OR (KW "shared decision making" OR "informed decision making" OR “patient decision making" OR "patient clinician communication" OR "informed consent")) |
| #3 | (TI "decision support*" OR "decision aid*" OR "decision tool*"OR "decision support system*") OR (AB "decision support*" OR "decision aid*" OR "decision tool*"OR "decision support system*" OR "decision support technique*") RO (KW "decision support*" OR "decision aid*" OR "decision tool*"OR "decision support system*" OR "decision support technique*")) |
| #4 | ((TI "Patient experience*" OR "patient perception*" OR "patient view*" OR "patient preference*" OR "patient satisfaction") OR (AB "Patient experience*" OR "patient perception*" OR "patient view*" OR "patient preference*" OR "patient satisfaction") OR (KW "Patient experience*" OR "patient perception*" OR "patient view*" OR "patient preference*" OR "patient satisfaction")) |
| #5 | #1 AND #2 AND #3 |
| #6 | #5 AND #4 |
| #7 | ((TI "vulnerable population*" OR "vulnerable person*" OR “vulnerable patient*" OR "vulnerable group*") OR AB ("vulnerable population*" OR "vulnerable person*" OR “vulnerable patient*" OR "vulnerable group*") OR (KW "vulnerable person*" OR "vulnerable patient*" OR "vulnerable group*")) |
| #8 | ((TI "middle income countr*" OR "low income countr*” OR “low to middle income countr*” OR "developing countr*") OR ( AB "middle income countr*" OR "low income countr*” OR “low to middle income countr*” OR "developing countr*") OR KW ("middle income countr*" OR "low income countr*” OR “low to middle income countr*” OR "developing countr*")) |
| #9 | #5 AND #7 |
| #10 | #5 AND #8 |
| **SCOPUS** | |
| #1 | (TITLE-ABS-KEY (cancer* OR oncolog* OR tumor* OR tumour* OR malignanc* OR neoplasm*) |
| #2 | (TITLE-ABS-KEY (“Shared decision making" OR "Informed decision making" OR "Patient decision making" OR "patient clinician communication" OR "informed consent ") OR (TITLE-ABS-KEY ("patient engagement" OR "patient involvement” OR "patient participation”)) |
| #3 | TITLE-ABS-KEY ("decision support*" " OR decision aid*" OR "decision tool*" OR "decision support system*" OR "decision support technique*") |
| #4 | TITLE-ABS-KEY ("Patient experience*" OR "patient perception*" OR "patient view*" OR "patient preference*" OR "patient satisfaction") |
| #5 | #1 AND #2 AND #3 |
| #6 | #5 AND #4 |
| #7 | TITLE-ABS-KEY ("Developing Countries" OR "low to middle income countr*" OR "middle income countr*" OR "low income countr*") |
| #8 | TITLE-ABS-KEY ("vulnerable population*" OR "disadvantaged population*" OR "under-served population*") |
| #9 | #5 AND #7 |
| #10 | #5 AND #8 |
| **Proquest** | |
| #1 | title(cancer* OR oncolog* OR tumor* OR tumour* OR malignanc* OR neoplasm*) OR abstract(cancer* OR oncolog* OR tumor* OR tumour* OR malignanc* OR neoplasm*) OR mainsubject(cancer* OR oncolog* OR tumor* OR tumour* OR malignanc* OR neoplasm*) |
| #2 | title("Shared decision making" OR "Informed decision making" OR "Patient decision making" OR "patient clinician communication" OR "informed consent" OR "patient engagement" OR "patient involvement" OR "patient participation") OR abstract("Shared decision making" OR "Informed decision making" OR "Patient decision making" OR "patient clinician communication" OR "informed consent" OR "patient engagement" OR "patient involvement" OR "patient participation") OR mainsubject("Shared decision making" OR "Informed decision making" OR "Patient decision making" OR "patient clinician communication" OR "informed consent" OR "patient engagement" OR "patient involvement" OR "patient participation") |
| #3 | title("decision support*" " OR decision aid*" OR "decision tool*" OR "decision support system*" OR "decision support technique*") OR abstract("decision support*" " OR decision aid*" OR "decision tool*" OR "decision support system*" OR "decision support technique*") OR mainsubject("decision support*" " OR decision aid*" OR "decision tool*" OR "decision support system*" OR "decision support technique*") |
| #4 | title("Patient experience*" OR "patient perception*" OR "patient view*" OR "patient preference*" OR "patient satisfaction") OR abstract("Patient experience*" OR "patient perception*" OR "patient view*" OR "patient preference*" OR "patient satisfaction") OR mainsubject("Patient experience*" OR "patient perception*" OR "patient view*" OR "patient preference*" OR "patient satisfaction") |
| #5 | #1 AND #2 AND #3 |
| #6 | #5 AND #4 |
| #7 | title("vulnerable population*" OR "vulnerable person*" OR “vulnerable patient*" OR "vulnerable group*" OR "disadvantaged population*" OR "under-served population*") OR abstract("vulnerable population*" OR "vulnerable person*" OR “vulnerable patient*" OR "vulnerable group*" OR "disadvantaged population*" OR "under-served population*") OR mainsubject("vulnerable population*" OR "vulnerable person*" OR “vulnerable patient*" OR "vulnerable group*" OR "disadvantaged population*" OR "under-served population*") |
| #8 | title("Developing Countries" OR "low to middle income countr*" OR "middle income countr*" OR "low income countr*") OR abstract("Developing Countries" OR "low to middle income countr*" OR "middle income countr*" OR "low income countr*") OR mainsubject("Developing Countries" OR "low to middle income countr*" OR "middle income countr*" OR "low income countr*") |
| #9 | #5 AND #7 |
| #10 | #5 AND #8 |
