# Supplementary material for: A mixed methods systematic review of cancer treatment decision-making in vulnerable populations
Source: Patient Educ Couns. Author manuscript; Available in PMC 2026 Jul 30. (PMC13419551; doi:10.1016/j.pec.2026.109491)
Supplement: 2 [file NIHMS2189791-supplement-2.docx]

**Supplementary Table A.2. Critical appraisal outcomes**

| Author (year) | Q1 | Q2 | Q3 | Q4 | Q5 | Q6 | Q7 | Q8 | Q9 | Q10 | Q11 | Q12 | Q13 | Score: n (%) |
| --- | --- | --- | --- | --- | --- | --- | --- | --- | --- | --- | --- | --- | --- | --- |
| **Randomised controlled trial** |  |  |  |  |  |  |  |  |  |  |  |  |  |  |
| AlSagheir, AI (2020) | Y | Y | Y | N | N | ? | ? | Y | Y | Y | ? | Y | Y | 8 (62) |
| Berry, DL (2013) | Y | Y | Y | N | Y | ? | Y | Y | Y | Y | Y | Y | Y | 10 (77) |
| Berry, DL (2018) | Y | Y | Y | N | ? | N | Y | Y | ? | Y | Y | Y | Y | 8 (62) |
| Diefenbach, MA (2018) | ? | N | Y | N | N | Y | Y | Y | ? | Y | Y | Y | Y | 8 (62) |
| Durand, M-A (2020) | Y | ? | ? | Y | ? | ? | ? | Y | Y | Y | Y | Y | Y | 8 (62) |
| Jalil, NB (2022) | Y | Y | Y | ? | Y | ? | Y | Y | Y | Y | ? | Y | Y | 10 (77) |
| Jibaja-Weiss, ML (2011) | Y | ? | Y | ? | ? | ? | Y | Y | ? | Y | ? | N | Y | 6 (46) |
| Joshi, S (2023) | Y | ? | Y | ? | ? | ? | Y | Y | Y | Y | ? | Y | Y | 8 (62) |
| Negarandeh, R (2023) | Y | Y | Y | ? | ? | ? | Y | Y | Y | Y | ? | Y | Y | 9 (69) |
| Tilburt, JC (2022) | Y | N | Y | ? | ? | ? | Y | Y | Y | Y | ? | Y | Y | 8 (62) |
| Wyld, L (2021) | Y | ? | Y | Y | N | Y | Y | Y | Y | Y | ? | Y | Y | 10 (77) |
| Yen, RW (2020) | Y | N | ? | ? | N | ? | Y | Y | Y | Y | Y | Y | Y | 8 (62) |
| **Analytical cross-sectional** |  |  |  |  |  |  |  |  |  |  |  |  |  |  |
| Li , KD (2021) | Y | Y | Y | Y | Y | Y | Y | Y |  |  |  |  |  | 8 (100) |
| **Quasi-experimental** |  |  |  |  |  |  |  |  |  |  |  |  |  |  |
| Wang, S., et al. (2022). | Y | Y | Y | Y | Y | Y | Y | Y | Y |  |  |  |  | 8 (89) |
| **Qualitative studies** |  |  |  |  |  |  |  |  |  |  |  |  |  |  |
| Alam, S (2016). | N | Y | Y | N | Y | N | N | Y | Y | Y |  |  |  | 6 (60) |
| Bamidele, OO (2021) | N | Y | Y | Y | Y | Y | Y | Y | Y | Y |  |  |  | 9 (90) |
| Burton, M (2015) | N | Y | Y | Y | Y | N | N | Y | Y | Y |  |  |  | 8 (80) |
| Durand, MA (2016) | N | Y | Y | Y | Y | Y | N | Y | Y | Y |  |  |  | 8 (80) |
| McVea, KISP 2001 | Y | Y | Y | Y | Y | N | N | Y | ? | Y |  |  |  | 7 (70) |
| Michel, J (2021) | Y | Y | Y | Y | Y | Y | N | Y | Y | Y |  |  |  | 9 (90) |
| Pan, S (2022) | N | Y | Y | Y | Y | N | N | Y | Y | Y |  |  |  | 7 (70) |
| Shaw, J (2015) | N | Y | Y | Y | Y | N | N | Y | Y | Y |  |  |  | 7 (70) |
| Sheppard, VB (2008) | Y | Y | Y | Y | Y | N | N | Y | Y | Y |  |  |  | 8 (80) |
| Sheppard, VB (2010) | Y | Y | Y | Y | Y | N | N | Y | Y | Y |  |  |  | 8 (80) |
| Wong, J-J (2011) | Y | N | Y | Y | N | Y | N | Y | Y | Y |  |  |  | 7 (70) |
| **Mixed Method Study** |  |  |  |  |  |  |  |  |  |  |  |  |  |  |
| Huang, S (2023) | N | Y | Y | Y | Y | N | N | Y | Y | Y |  |  |  | 7 (70) |

Abbreviations: Y = yes, N = no, NC = not clearly reported

| **Critical appraisal tools** |
| --- |
| **Randomised controlled trials.**  Q 1: Was true randomization used for assignment of participants to treatment groups  Q 2: Was allocation to treatment groups concealed? (Those allocating groups were blinded)  Q 3: Were treatment groups similar at the baseline? (Note for continuous variables examine means, not only p values)  Q 4: Were participants blind to treatment assignment?  Q 5: Were those delivering treatment blind to treatment assignment? (Doctors treating patients)  Q 6: Were outcomes assessors blind to treatment assignment? (Interviewers administering questionnaires)  Q 7: Were treatment groups treated identically other than the intervention of interest? (Check if other exposures may have had an effect)  Q 8: Was follow up complete and if not, were differences between groups in terms of their follow-up adequately described and analysed? (Not only numbers and proportions, also reasons and if different between groups, was it analysed?)  Q 9: Were participants analysed in the groups to which they were randomized? (Intention to treat analysis, analysed based on group and not whether intervention was used or not)  Q 10: Were outcomes measured in the same way for treatment groups? (Same instruments, timing, procedures and instructions?)  Q 11: Were outcomes measured in a reliable way? (Number of raters, training, inter-rater reliability (reported in the study itself), not validity of instruments.  Q 12: Was appropriate statistical analysis used? (Appropriate statistical tests, power analysis, effect sizes and assumptions of tests respected)  Q 13: Was the trial design appropriate (e.g., parallel, crossover, cluster, step-wedged) and any deviations from the standard RCT design accounted for in the conduct and in an analysis of the trial? |
| **Analytical cross-sectional studies**  Q1: Were the criteria for inclusion in the sample clearly defined?  Q2: Were the study subjects and the setting described in detail?  Q3: Was the exposure measured in a valid and reliable way?  Q4: Were objective standard criteria used for measurement of the condition?  Q5: Were confounding factors identified?  Q6: Were strategies to deal with the confounding factors stated?  Q7: Were outcomes measured in a reliable way?  Q8: Was appropriate statistical analysis used? |
| **Quasi-experimental studies**  Q 1: Is it clear in the study what is the 'cause' and what is the 'effect' (i.e. there is no confusion about which variable comes first)?  Q 2: Were the participants included in any comparisons similar?  Q 3: Were the participants included in any comparisons receiving similar treatment/care, other than the exposure or intervention of interest?  Q 4: Was there a control group?  Q 5: Were there multiple measurements of the outcome both pre and post intervention exposure?  Q 6: Was follow up complete and if not, were differences between groups in terms of their follow up adequately described and analysed?  Q 7: Were the outcomes of participants included in any comparisons measured in the same way?  Q 8: Were outcomes measured in a reliable way?  Q 9: Was appropriate statistical analysis used? |
| **Qualitative studies**  Q 1. Is there congruity between the stated philosophical perspective and the research methodology?  Q 2. Is there congruity between the research methodology and the research question or objectives?  Q 3. Is there congruity between the research methodology and the methods used to collect data?  Q 4. Is there congruity between the research methodology and the representation and analysis of data?  Q 5. Is there congruity between the research methodology and the interpretation of results?  Q 6. Is there a statement locating the researcher culturally or theoretically?  Q 7. Is the influence of the researcher on the research and vice versa addressed?  Q 8. Are participants and their voices adequately represented?  Q 9. Is the research ethical according to current criteria or, for recent studies, is there evidence of ethical approval by an appropriate body?  Q 10. Do the conclusions drawn in the research report flow from the analysis or interpretation of the data? |
