# Supplementary material for: A mixed methods systematic review of cancer treatment decision-making in vulnerable populations
Source: Patient Educ Couns. Author manuscript; Available in PMC 2026 Jul 30. (PMC13419551; doi:10.1016/j.pec.2026.109491)
Supplement: 3 [file NIHMS2189791-supplement-3.docx]

**Supplementary Table 3. Synthesised findings and themes derived from the qualitative papers**

| **Synthesized Finding 1** | **Categories** | **Findings** |
| --- | --- | --- |
| Patients differed in terms of their information needs and accessing sources of information, desired content and format. | Information need | **HIC** |
|  |  | **Information Needs Related to Decision Making for Breast Cancer Treatment (Wong 2011)**  *she wanted to know “what it is and what your options are in a very straightforward way. I don’t think you should be kept wondering.”* |
|  |  | **Information needs related to their breast cancer diagnosis** (**Wong 2011)**  *interested in understanding how physicians interpret the aggressiveness of certain cancer types after her physician told her that “the cancer cells of a tumor grow very slowly so that waiting” would not be serious concern* |
|  |  | **Perceptions (Sheppard 2008)**  *‘I did not have much information about cancer. That word scares you and is synonym of death. So obviously if you don’t have information, you don’t know what treatments there are.’*  *'Since I got cancer they never talked about chemotherapy, we [her family] did not know what it was.’* |
|  |  | **Access to Information: too much or not enough?** (**Shaw 2015)**  *“I feel more secure . . . he (doctor) explain it very clearly, very detailed. So I will be well prepared.” (047, Cantonese patient).*  *“I asked him (doctor) how long I will live. . .. The doctor won’t answer me. He said ‘you keep on taking the medication’.” (023, Cantonese patient)* |
|  |  | **LMIC** |
|  |  | **Theme I: less involved in treatment decision‑making (Lack of medical knowledge)** (**Pan 2022)**  *“According to what I’ve heard, this kind of sickness is rather frequent among the elderly. My PSA (prostate-specific antigen) level has been elevated for a lengthy period, but I have no idea what this indication implies precisely, and I have no idea where to seek genuine assistance.”*  *“I am an illiterate with limited knowledge of characters. I was unaware of the exact type of disease I had contracted. So, I am not sure which is better.”* |
|  |  | **Theme 2. The right to be informed of the disease condition and to choose treatment options (Patients believe that they should know the true facts of the disease)** (**Pan 2022)**  *“I genuinely want to be involved in my disease treatment, but no one informed me, and I’m still puzzled. What alternatives do I have for treatment? Which is the more radical and which is the more conservative? … So that I am aware of what to expect. … In any case, whatever kind of therapy is more suited for me now and in the future? I still want more information.”* |
|  |  | **Theme 3. Future consideration and advance care planning (Advance care planning is poorly understood)** (**Pan 2022)**  *“Because I am unfamiliar with advance care planning, I have no plan for myself.”* |
|  | Information gathering | **HIC** |
|  |  | **Access to Information: too much or not enough?** (**Shaw 2015)**  *“No the doctor first tell me you need this. . . and then I asked the other doctors and they say this is the best so of course I come back and say I want to do it because they all say it’s the best. I don’t know what’s the best they do” (Arabic patient)*  *“You can find it on the website in Chinese oncology hospital what you should take [treatment] they had it all” (Cantonese patient)* |
|  |  | **Seeking out those who speak my language** (**Shaw 2015)**  *“I made calls. . . I wanted to find one who spoke Chinese, its better for communication. Then I found Dr XXX speaks Chinese and his receptionist also spoke Chinese, then we could communicate.” (Cantonese patient)* |
|  |  | **Information Needs Related to Decision Making for Breast Cancer Treatment** (**Wong 2011)**  *connected with another cancer survivor, who as she expressed, “explained everything to me and [answered] any questions I had.”* |
|  |  | **English-Speaking Latinos Feel More Comfortable Utilizing Digital Technology for Information Search** (**Michel 2021)**  *I pay attention to what the urologist says and that is enough information for me. He wants to help me and I listen. (61-year-old Mexican Spanish speaker, 8 years of education)* |
|  |  | **Getting empowerment through information-seeking (Bamidele, 2021)**  *… so they (medical team) gave me all the options of cure, so I told them I need time to you know go through, I took about two weeks and made my research on the internet, going to libraries, talking to a few friends who are doctors, you know, I gathered as much information as I can and then I sat down and I analysed the situation, analysed the side-effects of all the treatment and none of them was equally suitable, but at least I could get close to the brachytherapy… (Mr Dave, BA, 62)* |
|  |  | **LMIC** |
|  |  | **Theme 2: disease information acquisition (Information sources included doctors, family members, patients, mobile phone access, and checklists, Preference toward information acquisition, Information filtering (Requested by family)) (Huang 2023)**  *“…it collapsed when I first knew it and I asked my relatives and friends about it. My family was very worried.”*  *“The doctor said clearly and the effect is very obvious. We checked it online and learned something”*  *‘As soon as I have a question, I'll ask the doctor. Doctors try their best to answer”* |
|  | Information format and presentation | **HIC** |
|  |  | **English-Speaking Latinos Feel More Comfortable Utilizing Digital Technology for Information Search** (**Michel 2021)**  *I use [cellphone] to get into the web, to look for information within YouTube, that’s how I do my research like for cancer for my prostate. (55-year-old Mexican English speaker, 12 years of education)*  *I do not use any computer of any sort. I only use the phone and actually I do not know how to use it that well. . .. I do not know how to operate a smartphone. (57-year-old Mexican Spanish speaker, 6 years of education)* |
|  |  | **Information - use, preferred content, and format (Burton 2015)**  *when you’ve read about six pages you put it down....and as you get older them six pages you never get past because you keep reading the same ones. By the next day you’ve forgotten what it’s saying. Yeah, that’s good that because it gives you all your questions?’ (94 yrs)*  *“I’ve got it, [internet] but I don’t bother about it very much ... No, it would be the last thing I’d do [go to the internet]”. (80 yrs)* |
|  |  | **Does written information facilitate discussions?** (**Shaw 2015)**  *“I can read English, but I don’t understand the meaning of the words I have to take out the dictionary every time I come from the doctor’s. I just sit there and say yes, yes but I leave his consultation and I didn’t understand most things. I find it hard” (094 Arabic patient).*  *“There was no ‘‘ANY WRITTEN REPORT SAYING WHAT IT WAS!!’’(the patient stressed this in English). Until now I still don’t have. Never. . .Never anything in writing.” (013, Mandarin patient)* |
|  |  | **Information Needs Related to Decision Making for Breast Cancer Treatment** (**Wong 2011)**  *her surgeon was helpful because he had explained “exactly what he was going to do and drew a simplified diagram.”* |
|  |  | **Purpose of the encounter decision aids. (Preparing and informing patients, Complete overview of information)** (**Alam 2016)**  *“I think those [FAQs] are great questions […], and this would’ve helped me a lot.”* |
|  |  | **Benefits of the encounter decision aids (Pictorial superiority, Realistic portrayal of the treatment process, Language accessibility, Promotes engagement in decision making)** (**Alam 2016)**  *“I sometimes think when people come in and they have someone talking to them, they don't always hear everything they say, and I think seeing a visual sometimes is more helpful.”* |
|  |  | **Pictorial superiority** (**Durand 2016)**  *And sometimes when people explain things it sounds different but to see the pictures and to have an idea of what actually was happening I think would help a lot of people. (Patient, 60)* |
| **Synthesized Finding 2** | **Categories** | **Findings** |
| Decision making is a process that starts before the treatment decision consultation, requires effort from the patient to consider options under stressful circumstances, often making choices that are not only based on clinical information. | Process and timing | **HIC** |
|  |  | **Perceptions (Sheppard 2008)**  *‘when the doctor gets you by surprise you believe in that doctor and you don’t have a chance to check with another doctor. At the time you’re so tense with the news they gave you that you don’t see another escape and the only one is that one. That’s the only one. So you do what the doctor tells you. You don’t get a chance to think about it. Once you get the news you have cancer, you get confused and don’t know what to do.’* |
|  |  | **Time pressure to decide** (**McVea 2001)** *‘things have to be decided too fast’....‘lack of knowledge’ ... ‘just didn’t feel sure’* |
|  |  | **Enablers: Positive Existential Negative** (**Sheppard 2010)**  *‘The problem is that you just don’t get the right information at the right time.’* |
|  |  | **Feasibility of the encounter decision aids. (Post-diagnosis, Before or during the surgical consultation, Beneficial for all)** (**Alam 2016)**  *“So, if it was given to me at the time of diagnosis and then I had the appointment with the surgeon to discuss all of this, then it would give me a better idea of what questions to ask.”* |
|  |  | **Before or during the consultation** (**Durand 2016)**  *I’d like to have something like this in the mail before I go and see my surgeon. (Patient, 74)* |
|  |  | **LMIC** |
|  |  | **Theme 1: the dynamic change of decision‑making mode. (From passive to shared, From family-patient-doctor sharing to patient-doctor sharing.) (Huang 2023)**  *…at first, I didn’t care much. Later, there might be. one point, most of which were discussed by children and doctors. At first the kid didn’t tell me much about it. Just listen to the doctor. Slowly I know. He didn’t have time to change my pockets after the surgery, and finally I did. Last year in your department for treatment, he can’t come with me every time, I don’ t need him to accompany. I can provide my own signature and medicine is also my own to buy* |
|  | Work of deliberation on options/choices | **HIC** |
|  |  | **Evaluation (Sheppard 2008)**  *the navigator helped her to 'get in the habit of writing questions and reviewing them before seeing her doctor.’* |
|  |  | **Beneficial for all** (**Durand 2016)**  *Some people don’t like to sit and read stuff and it’s easier with the picture grid to go through everything. (Patient, 60)* |
|  |  | **Enablers: Positive Existential Negative** **(Sheppard 2010)**  *‘You know, they always ask [if I have anything to say, and I never have anything to say so it’s mostly my fault.’* |
|  |  | **Perceptions: Positive Existential Negative** **(Sheppard 2010)**  *‘I know what [chemotherapy] did to my husband. It took all of his hair out.’* |
|  |  | **Preparing for upcoming treatments (Durand 2016)**  *In all the pages, it shows that you either take part of your breast or all of your breast, then it shows you how long it takes, then it shows you the radiation, so I know what’s coming…that was my fear, what is coming. (Patient, 74)*  *With the graphics of the mastectomy, I think that’s a great graphic to have there. I mean, that was one of those things I said, well, I want to see what it would look like if you do this and what it would look like if I did that, to give me an idea at that time. (Patient, 56)* |
|  |  | **LMIC** |
|  |  | **Theme 2. The right to be informed of the disease condition and to choose treatment options (3. Patient autonomy during treatment) (Pan 2022)**  *“Occasionally, I like to get comprehensive knowledge about the sickness to prepare ahead. … My wife and I co-managed a grocery store. Should we close the business to cover medical costs?”* |
|  |  | **Theme 3: SDM obstacles (Lack of knowledge)** (**Huang 2023)**  *I don’t think I should be involved in this process. I don’t know about pharmacology. I believe in doctors* |
|  |  | **Theme 3. Future consideration and advance care planning (Acceptance of advance care planning)** (**Pan 2022)**  *“Every man is born to die. I’m not interested in living in agony. I hope I can live out the remainder of my days happily, without causing distress to others.”* |
|  |  | **Theme 3: SDM obstacles (Insurance affects patients’ SDM choice)** (**Huang 2023)**  *We bought rural cooperative medical insurance last year, and breast insurance for 100 yuan in early years. The previous year we did not buy anything, and then sick. No way, we now buy the new rural cooperative medical insurance* |
|  | Reasons for choices | **HIC** |
|  |  | **Treatment decision making (Burton 2015)**  *I decided the years I’ve got left… I’m not messing about going into hospital…’ (95 yrs)*  *Right, I said,‘ let’s get rid of it, at my age,’ so I went for a full (mastectomy]. But if I [h]adn’t have had a [mastectomy] I’d have to have had radiotherapy...’ (75yrs)* |
|  |  | **Reason for treatment choice, cosmetics, family responsibility, financial** (**McVea 2001)**  *‘I’m raising a child that is two years old. I cannot be in and out of the hospital. I said I need all this done now’*  *‘I was just going to have to leave it [the cancer] go’. (had no insurance)*  *‘I guess it just comes down to cosmetically, you don’t want to be deformed’* |
|  |  | **Perceptions: Positive Existential Negative (Sheppard 2010)**  *‘I know what [chemotherapy] did to my husband. It took all of his hair out.’* |
|  |  | **Enablers (Sheppard 2008)**  *‘The decision to get chemotherapy was like either you do it or you do it. They don’t give you a chance whether you want to do it or not. But I think this decision is whether you want to live or not. It’s survival’.* |
|  |  | **Choosing a treatment option: Prioritising survival from the prostate cancer (Bamidele, 2021)**  *… when it was diagnosed as prostate cancer, my consultant then said, ‘what do you want to do you've got only a number of options’ and I said to him look, listen I've had all the children that I'm gonna have, I want it out and he said, ‘well you know we could do brachytherapy, we can put you on hormone’, I said no, I said I'm done (authoritatively) I said we're gonna go for, ahm we're gonna go for the prostatectomy I've researched it, I've done a far bit of research by now ah and I said BA, 50) we'll go for the Da Vinci ok fine… (BA, 50)* |
|  |  | **Choosing a treatment option: Reluctance to lose a ‘significant’ body part and function (Bamidele, 2021)**  *… so looking at all the options, the one that was less invasive and also can get you back to your fit quickly was the brachytherapy… (BA, 66)* |
| **Synthesised Finding 3** | **Categories** | **Findings** |
| Decision making consultations are affected by the style of clinician and patient interaction impacting on decision control, where patients sometimes adopted roles that they did not prefer, and preferred format of decision supports varied. | Clinician patient interaction | **HIC** |
|  |  | **Physician decision-making style: Informative** **(McVea 2001)**  *‘He didn’t tell me which [treatment] he thought I should do. He was perfectly objective'.*  *‘Everything was left up to me’.* |
|  |  | **Physician decision-making style: Reflective** **(McVea 2001)**  *One woman remembered her physician saying, ‘We’re all in this together’.* |
|  |  | **Physician decision-making style: Paternalistic (McVea 2001)**  *‘I didn’t really discuss very much of it. Dr.P. would tell me what he thought I should have done’.*  *'It was everything they wanted me to know, nothing that I needed to know. But I didn’t know this. I couldn’t ask these questions because I didn’t know what to ask until after it was over'*  *‘I think that was the worst part of my whole breast cancer surgery...Nobody was listening to me’.* |
|  |  | **The Presence of Paternalistic Patient-Physician Relationship Was Common** **(Michel 2021)**  *"He said, ‘I am going to get you all the test you need and see if I can add you for surgery soon' . . . the doctor was a little bit rough with the news". (66-year-old Mexican English-speaker, 11 years of education)*  *"I did not talk much, [the doctor] only told me to manage my food intake. . .. She only said to eat less. . .. She scolded me because I eat fried foods. . .. I could change clinics, but I am doing well with her. I have to respect the rules". (70-year-old Mexican Spanish speaker, 6 years of education)* |
|  |  | **Suggestions to Healthcare Professionals for Better Communication During Breast Cancer Diagnosis and Treatment (Wong 2011)**  *“medical people talk to us as if we know exactly what they are talking about...long explanations...they expect you to know.”*  *“Hi. How are you? Can I see your breast?” because they seemed more interested in her breast than her* |
|  |  | **Promotes engagement in decision-making** (**Durand 2016)**  *It leaves room for you to ask questions. I mean it puts things into your—into perspective for you to understand more and to be more comfortable asking your doctor, I think. (Patient, 68)* |
|  |  | **LMIC** |
|  |  | **Theme 3: SDM obstacles (Doctors’ lack of time)** **(Huang 2023)**  *Sometimes health professionals were so busy that they didn’t care about anything. I was sorry to ask you* |
|  | Preferred or perceived passive decision role | **HIC** |
|  |  | **Perceived role of the patient in decision-making (Shaw 2015)**  *“It’s up to the doctor from the very beginning to the end, whatever the doctor says we just follow. . . It leaves you no room for decision. I just feel that it’s not up to me to decide, I am not a professional doctor so I have to rely on the doctor, whatever he tells me to do” (Cantonese patient)*  *“For my case, the specialist never discussed with me, never took my opinion in consideration. . . The doctor won’t listen to you. It’s not that we didn’t want, and it’s not that we didn’t initiate to know more, I really wanted to know, I felt helpless, felt myself helpless. He ‘‘HOLD’’ the ‘‘POWER’’.” (Mandarin patient)* |
|  |  | **Women’s decision styles: passive, avoidant, panicked, rational (McVea 2001)**  *‘I just don’t fight the doctor.’* |
|  |  | **Treatment decision making** (**Burton 2015)**  *‘...you’re a bit gobsmacked [when they give you a choice] you don’t know what….’ well obviously, he deals with that all day and every day so I just said, ‘Well what do you advise? I mean what do you see these people for if not to take their advice?’ (81 yrs)* |
|  |  | **LMIC** |
|  |  | **Theme I: Less involved in treatment decision‑making (Passive decisional control) (Pan 2022)**  *“I have registered a specialist clinic, which is costly and precious. As a result, I would follow the expert’s advice.”*  *“Children have the final say in family matters. I am already a senior citizen. Allow my children to discuss these issues with doctors.”* |
|  | Preferred or perceived active decision role | **HIC** |
|  |  | **Treatment decision making** (**Burton 2015)**  *‘… I’d already made my mind up because I knew it was cancer…—you know in my own mind and made my mind up that I was having the breast taken off.’ (80 yrs)*  *‘He [Surgeon] seemed pleased with my decision.’ (76 yrs)* |
|  |  | **LMIC** |
|  |  | **Theme 2. The right to be informed of the disease condition and to choose treatment options (Patient autonomy during treatment)** **(Pan 2022)**  *“Since I have been in the hospital, I have been instructed to undergo various checks or tests. It’s as if I were a puppet. I want to be able to decide whether or not to do these checks.”* |
|  |  | **Theme 2. The right to be informed of the disease condition and to choose treatment options (Patients believe that they should know the true facts of the disease) (Pan 2022)**  *“I genuinely want to be involved in my disease treatment, but no one informed me, and I’m still puzzled. What alternatives do I have for treatment? Which is the more radical and which is the more conservative? … So that I am aware of what to expect. … In any case, whatever kind of therapy is more suited for me now and in the future? I still want more information.”* |
|  | Consultation decision support | **HIC** |
|  |  | **Enablers: Positive Existential Negative (Sheppard 2010)**  *'I have excellent communication with [providers]. They explain things. They take time with me. They always call me back. They’ve been very open about the prognosis.'* |
|  |  | **Nurturers: Positive Existential Negative** **(Sheppard 2010)**  *‘I had two of my daughters with me, and they asked a lot of questions. But since I’ve been going by myself, I [have] really felt neglected.’* |
| **Synthesised finding 4** | **Categories** | **Findings** |
| Patients experienced strong emotions following a cancer diagnosis that impacted on their ability to make decisions. Social, cultural and language factors influenced decision making, indicating the need for an enhanced psychosocial approach to decision support. | Emotional/Social impact of cancer and treatment decision making. | **HIC** |
|  |  | **Women’s decision styles: passive, avoidant, panicked, rational** **(McVea 2001)**  *‘Total panic and get rid of it as fast as possible’.*  *'depressed.... alone.... intimidated’.*  *‘[I]was kind of foggy for a while, just figuring what to do next...It took me until February to get it together’.* |
|  |  | **The impact of discovering breast cancer** **(Burton 2015)**  *I just kept saying, ‘Do what you’ve got to do, do what you’ve got to do’. We lost a daughter-in-law with breast cancer, she was only 26, and that’s 30 years ago... she would have still been alive if they’d have taken it off. (84yrs)*  *‘.. I thought, ‘... I’m going to die with this, so we’ll base it round that’. (85 yrs)* |
|  |  | **Patient emotional response to cancer – denial, fear, anger, sadness** **(McVea 2001)**  *‘The problem was I wasn’t deciphering [the information] because I was so afraid’.*  *‘He’s not talking about my body; he’s talking about somebody else’s body’.* |
|  |  | **Coming to terms with the prostate cancer diagnosis (Bamidele 2021)**  *…I was scared erm I'm a very practical man erm and I'm also the breadwinner, so my family is very reliant on me in terms of you know financial well-being… I was scared for them I was saying then how do I make provisions for them, what's the next steps… (BC, 51)* |
|  | Family, social, cultural influences and language barriers in decision making. | **HIC** |
|  |  | **Nurturers** **(Sheppard 2008)**  *‘Us as mothers we worry about everybody, our kids, our husbands, our work and the last one we think of are we. We have to be conscious that we are the heads of the family and we have to take care of ourselves’.* |
|  |  | **The Importance of Family Involvement and Their Role in Decision Making.** **(Michel 2021)**  *I do have family, but they don’t say much. They do not give me much information. . .. My family knows about the problem, but they only say for me to take care. Nothing else. (70-year-old Mexican Spanish speaker, 6 years of education)*  *I did not want people to know about it, only my wife. [regarding the decision-making process], I think I will do it alone. (57-year-old Mexican Spanish speaker, 6 years of education)*  *My wife did the research for me in books and [about] natural remedies. (55-year-old Mexican English speaker, 12 years of education)* |
|  |  | **Involvement of family in decision making** **(Shaw 2015)**  *“I told him (doctor) . . . ‘don’t open your mouth, talk with my husband, talk with my children, and have a meeting with all of them. . . Don’t inform me, I don’t want to know, I am happy this way. “(Arabic patient)* |
|  |  | **Cultural influences on decision making - Differing health concepts** **(Shaw 2015)**  *“I take Chinese herb, I asked my doctor. The doctor didn’t agree with me at the beginning. For example, during the chemotherapy, the doctor told me I shouldn’t take anything, but I felt it would be better. I took Ling-Zhi and shark bone powder now.” (Mandarin patient)* |
|  |  | **Cultural influences on decision making - Impact of religious beliefs** **(Shaw 2015)**  *“The doctor tells me to do something, you shouldn’t say ‘no, I won’t do it’, this is in His [Gods] name, whatever they say, we do.” (Arabic)* |
|  |  | **Language as a barrier to decision-making participation** **(Shaw 2015)**  *“I can read English but I don’t understand the meaning of the words I have to take out the dictionary every time I come from the doctor’s. I just sit there and say yes, yes but I leave his consultation and I didn’t understand most things. I find it hard” (Arabic patient).* |
|  |  | **Language as a barrier to decision-making participation. (Does written information facilitate discussions?)** **(Shaw 2015)**  *“I don’t understand English and everything they gave me was in English. No Greek person has spoken to me. Yes I have difficulties; I didn’t understand him in everything he said.” (Greek patient)* |
|  |  | **Language as a barrier to decision-making participation (Seeking out those who speak my language)** **(Shaw 2015)**  *“For us it is difficult. As Chinese in overseas, when we have a problem, we can’t express our symptoms, so we find an interpreter. There are so many interpreters, they phone in. They don’t say it right [the meaning of what is being said]. So often our message is not correctly delivered. The doctor doesn’t understand us” (Cantonese patient)* |
|  |  | **Enablers (Sheppard 2008)**  *‘My biggest worry was to be able to endure the chemo and continue taking care of my kids. I want to be with my family, which is the most important thing in my life, more than my life. If you get cured, you want to do it for your kids’* |
|  |  | **Choosing a treatment option: Medical treatment as a last resort (Bamidele, 2021)**  *… I was also in touch with an herbalist. I'm still in touch with him to tell you the truth and I thought well you know maybe I can use natural medicines to cure myself you know so I then decided to get in touch with him and other people from his kind and erm to see if I can use natural means to change my diet and you know to really make a difference… I was opting for it because I thought to myself maybe there is a connection to a longer life a natural life with herbs instead of synthetic drugs and especially with radiotherapy…. (BC, 69)* |
|  |  | **Choosing a treatment option: Prioritising masculine responsibilities to the family over own cancer treatment (Bamidele, 2021)**  *… and the oncologist spoke to me and said if ‘you come off this (treatment) you will die’, and my words to him was if I don't I will die, you need to understand my mental state right now… if you do not work something out for me, I am coming off, there is nothing you can do for me or anybody can do I am coming off, I know the consequences but if I don't, I'm gonna die so you have to understand where I am at, I need to earn my money, I need to be there for my family, I need to be able to provide for my family… I have to as a man look to my family ok I have to,… , if I am not able to turn around and have that ability to provide for myself then mentally it is destroying me, it will destroy me… (BC, 50)* |
|  |  | **LMIC** |
|  |  | **Theme 2: disease information acquisition. (Information filtering (Requested by family)) (Huang 2023)**  *My daughter said nothing. Then I asked the nurse, the nurse may be afraid I know, said she did not know* |
|  |  | **Theme 4: family member roles (Auxiliary support, Decision-making control)** (**Huang 2023)**  *Family members mainly took care of me. They also helped a lot, when deciding what to do to. They usually tell me what to pay attention to.*  *I don’t know how children and doctors negotiated. They let me treat here first, and then went back to surgery. I asked if I couldn’t do the surgery. The kid said no, I had to do the surgery. I do not have a choice.* |
|  |  | **Theme I: less involved in treatment decision‑making (Domination by family members)** (**Pan 2022)**  *“My family, relatives, and acquaintances are all aware of my sickness, yet I was an outlier.”*  *“My son informed me last week that I would be having surgery, without providing any other information. … If I have surgery, would there be several complications, such as urinary incontinence? How long will recovery take? Additionally, I am concerned about sexual dysfunction. I want to sustain sexual function. However, my wife was the one who made major domestic decisions. I was excluded since my family believed I was helpless due to my vulnerability. And this time, it was my wife and children who spoke with the doctor.”* |
|  |  | **Theme 2. The right to be informed of the disease condition and to choose treatment options. (Sociocultural influences)** **(Pan 2022)**  *“My son is fully aware of my disease and has overseen all aspects of it, including treatment, surgery, and so on. He assured me that I should not be concerned. My blood pressure is unstable.”*  *“I assumed the diagnosis would be negative, and the doctor advised me to wait outside the door.”* |
|  |  | **Theme 3. Future consideration and advance care planning (Fewer future concerns) (Pan 2022)**  *“Each time, it is my children who see the doctor. When I inquired about my ailment, they said, ‘not too terrible.’ However, if I want to speak with the doctor alone, there are usually several patients there. Then I gave up and abandoned it, not to mention the future.”*  *“Since I became ill, my family members have avoided discussing this subject in front of me out of fear of making me upset. Indeed, I am concerned and saddened. I’m not interested in discussing the future.”* |
|  | Strategies for socio-emotional support | **HIC** |
|  |  | **Suggestions to Healthcare Professionals for Better Communication During Breast Cancer Diagnosis and Treatment** **(Wong 2011)**  *it would have been helpful to meet with a social worker or psychologist to help them come to terms with “stresses they are going through.”* |
|  |  | **Evaluation (Sheppard 2008)**  *‘having a person that went through the same thing transmitted a sense of confidence and hope'.* |
|  |  | **Satisfaction with survivor coaches (Sheppard 2010)**  *‘I appreciated speaking with her. She made me feel quite at ease in the face of a difficult situation. She gave me hope.’* |
